# Ectoparasite and bacterial population genetics and community structure indicate extent of bat movement across an island chain

**DOI:** 10.1017/S0031182024000660

**Published:** 2024-06

**Authors:** Clifton D. McKee, Alison J. Peel, David T. S. Hayman, Richard Suu-Ire, Yaa Ntiamoa-Baidu, Andrew A. Cunningham, James L. N. Wood, Colleen T. Webb, Michael Y. Kosoy

**Affiliations:** 1 Department of Epidemiology, Johns Hopkins Bloomberg School of Public Health, Baltimore, MD, USA; 2 Centre for Planetary Health and Food Security, Griffith University, Nathan, QLD, Australia; 3 Molecular Epidemiology and Public Health Laboratory (mEpiLab), Infectious Disease Research Centre, Hopkirk Research Institute, Massey University, Palmerston North, New Zealand; 4 School of Veterinary Medicine, University of Ghana, Accra, Ghana; 5 Centre for Biodiversity Conservation Research, University of Ghana, Accra, Ghana; 6 Department of Animal Biology and Conservation Science, University of Ghana, Accra, Ghana; 7 Institute of Zoology, Zoological Society of London, Regent's Park, London, UK; 8 Disease Dynamics Unit, Department of Veterinary Medicine, University of Cambridge, Cambridge, UK; 9 Graduate Degree Program in Ecology, Colorado State University, Fort Collins, CO, USA; 10 Department of Biology, Colorado State University, Fort Collins, CO, USA; 11 KB ONE Health, LLC, Fort Collins, CO, USA

**Keywords:** Africa, *Bartonella*, bat flies, Chiroptera, endosymbionts, host–microbe interactions, Nycteribiidae, phylogeography

## Abstract

Few studies have examined the genetic population structure of vector-borne microparasites in wildlife, making it unclear how much these systems can reveal about the movement of their associated hosts. This study examined the complex host–vector–microbe interactions in a system of bats, wingless ectoparasitic bat flies (Nycteribiidae), vector-borne microparasitic bacteria (*Bartonella*) and bacterial endosymbionts of flies (*Enterobacterales*) across an island chain in the Gulf of Guinea, West Africa. Limited population structure was found in bat flies and *Enterobacterales* symbionts compared to that of their hosts. Significant isolation by distance was observed in the dissimilarity of *Bartonella* communities detected in flies from sampled populations of *Eidolon helvum* bats. These patterns indicate that, while genetic dispersal of bats between islands is limited, some non-reproductive movements may lead to the dispersal of ectoparasites and associated microbes. This study deepens our knowledge of the phylogeography of African fruit bats, their ectoparasites and associated bacteria. The results presented could inform models of pathogen transmission in these bat populations and increase our theoretical understanding of community ecology in host–microbe systems.

## Graphical abstract

**Figure:**
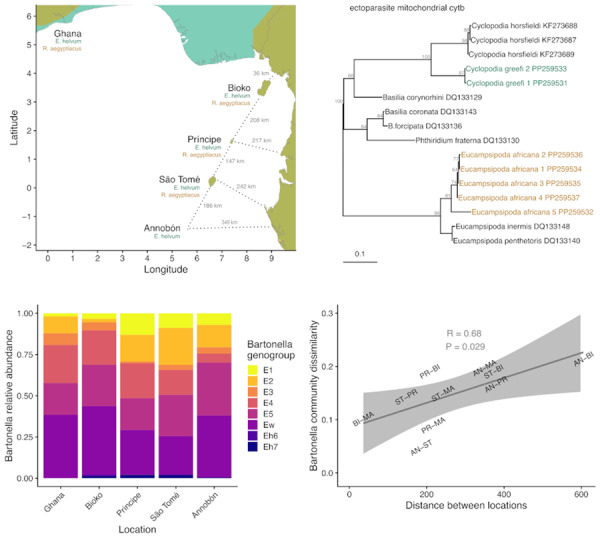

## Introduction

A key question in biology is how populations and communities of organisms are structured across space and time. This question is united in the theory of population genetics and community ecology *via* the theme of movement (Vellend, 2010), either gene flow *via* the movement of individuals (and associated alleles) between populations or the movement of species between communities. Holding all other processes constant, the frequency of movement produces results ranging from panmixia or community homogeneity to the complete fixation of alleles or of species. While organismal movement is challenging to measure directly at scale, researchers can rely on molecular genetic tools to infer the movement of individuals and alleles between populations. However, movements that do not lead to reproduction cannot be detected from such genetic data. A potential solution is to explore the population genetics of mutualistic or parasitic organisms to shed light on the total degree of connectedness of the host populations, including both reproductive and non-reproductive movements (Nieberding and Olivieri, 2007).

**Figure 1.**
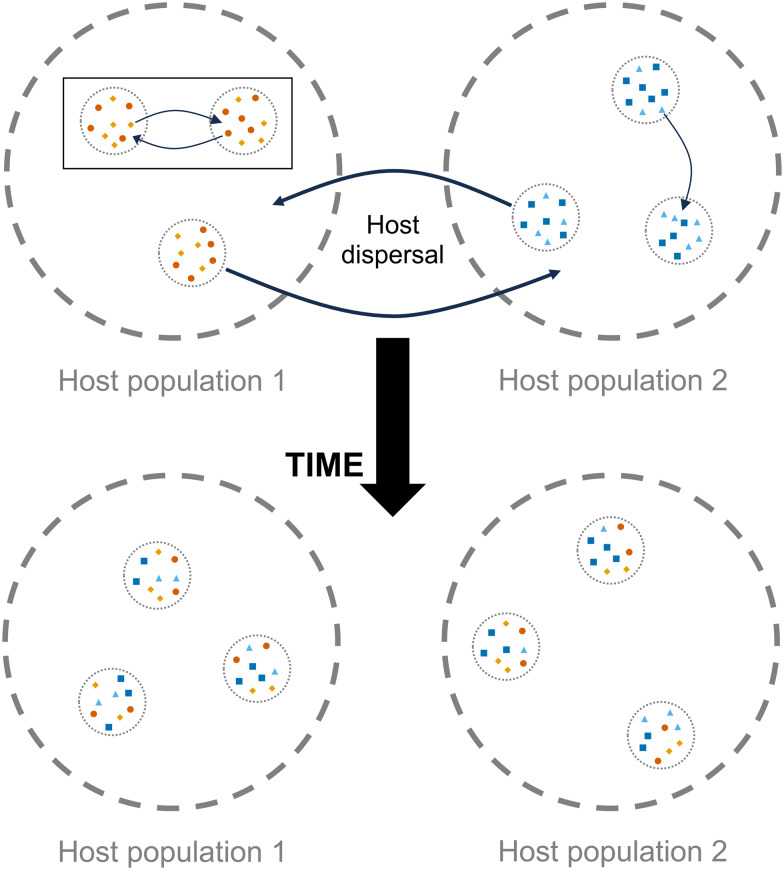
Conceptual diagram for microbial community dynamics among host populations. Microbe species (coloured dots) exist within hosts (dotted circles), which in turn, exist within host populations (dashed circles). Microbes are transmitted among hosts within a population (inset box). Over time, dispersal of infected host individuals (or vectors) between populations may alter the frequency of alleles or species within microbe communities. Sufficient dispersal between host populations may lead to homogeneous microbial communities.

Successful examples showing that parasites can provide a refined understanding of host movement come from human ecology (Falush *et al*., 2003; Holmes, 2004) and notable wildlife studies (Nieberding *et al*., 2004; Biek *et al*., 2006; Criscione *et al*., 2006; Lee *et al*., 2012). While these examples have focused on subpopulation structure in individual host and parasite species, similar patterns might be observable at higher levels of ecological organization, such as the community structure of mutualistic and parasitic microbes (Mihaljevic, 2012; Seabloom *et al*., 2015). In this form of analysis, the agents under consideration are not alleles moving between populations but rather individuals of distinct species moving between infracommunities of microbes within hosts, potentially resulting in varying relative abundance of microbial species across host populations (Fig. 1). Whether assessing movement at the scale of microbe population genetics or community species composition, the ability to detect structure depends on the choice of appropriate molecular markers and the life history of the microbe (Jarne and Théron, 2001; Nieberding and Olivieri, 2007). Microbes that rely on vertical transmission, or horizontal transmission without a free-living stage or alternative hosts, would be expected to be ideal proxies for associating population or community structure with host movement since the movement of such microbes is intimately tied to the behaviour of a single host species (Wirth *et al*., 2005; Nieberding and Olivieri, 2007).

In the case of microbes with multiple potential hosts, particularly vector-borne microparasites, any structure observed might be challenging to interpret. It has been hypothesized that the population structure of a multi-host parasite should reflect the movement patterns of its most vagile host, since any structure generated by another isolated host will be overwhelmed by frequent dispersal events facilitated by the vagile host (Jarne and Théron, 2001). Yet, this expectation might be complicated by the nested levels of dependence in vector-borne microparasite systems, wherein the microparasite is dependent on the vector for transmission between hosts, and the vector, being a parasite itself, is dependent on the host for completion of its own life cycle. Previous studies of host-restricted, ectoparasitic vectors and associated microparasites have shown that vectors can show less population structure than their hosts (van Schaik *et al*., 2018), and either no genetic structure in the microparasites (Levin and Parker, 2013) or poor correlation between the differentiation in microparasite subpopulations with the structure apparent in their hosts or vectors (Witsenburg *et al*., 2015). It is possible that the low genetic differentiation in vector-borne microparasites is due to the additive effect of host and vector movements (Witsenburg *et al*., 2015), facilitating high levels of gene flow in microparasite populations. Additional examinations of population and community structure in hosts, vectors and their associated mutualistic and parasitic microbes are needed to find general patterns across systems.

**Figure 2.**
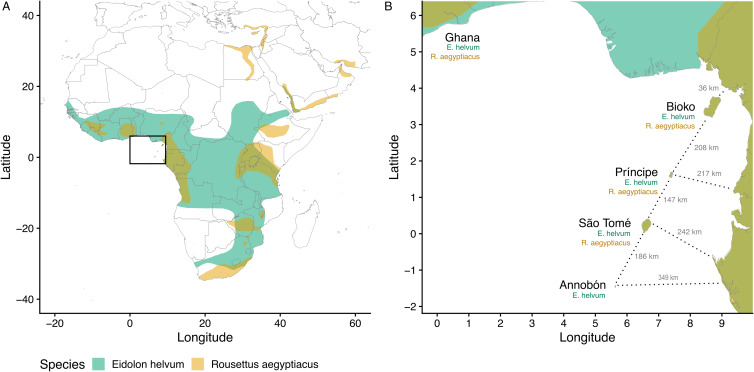
Map of study area in West Africa (A), islands in the Gulf of Guinea (B). Axis values are in degrees latitude and longitude. Segments for estimating the shortest distance between islands and the mainland are shown as dotted lines. Bat geographic ranges were retrieved from the IUCN Red List website (https://www.iucnredlist.org/), with modifications to display the occurrence of species on the Gulf of Guinea islands.

The system chosen for the current study is especially suitable for this type of investigation because of the contained nature of the focal host populations and the traits of the parasites. This study focuses on 2 species of fruit bat (Chiroptera: Pteropodidae), *Eidolon helvum* and *Rousettus aegyptiacus*; their ectoparasitic bat flies (Diptera: Nycteribiidae), *Cyclopodia greefi* and *Eucampsipoda africana*; and 2 taxa of bacteria, the genus *Bartonella* (*Alphaproteobacteria*: *Hyphomicrobiales*) and the order *Enterobacterales* (*Gammaproteobacteria*). The bat species are distributed across Africa and can be found on several isolated islands in the Gulf of Guinea (Fig. 2). Studies on both bat species have found that island populations are genetically distinct from each other and from mainland populations. Specifically, *E. helvum* from Annobón is a distinct subspecies (*E. helvum annobonense*) and individuals are significantly smaller than those present on the mainland and the other Gulf of Guinea islands (Juste *et al*., 2000). Similarly, *R. aegyptiacus* from São Tomé and Príncipe are genetically and morphologically distinct from each other and from populations on Bioko and the mainland and are recognized as distinct subspecies (*R. aegyptiacus princeps* and *R. aegyptiacus tomensis*) (Juste and Ibáñez, 1993; Juste *et al*., 1996; Stribna *et al*., 2019). Two bat fly species are obligate ectoparasites specific to their host species, *C. greefi* to *E. helvum* and *E. africana* to *R. aegyptiacus* (Theodor, 1955, 1957). These haematophagous flies spend almost their entire lives on their bat hosts, with gravid females only leaving to deposit a single third-instar larva on the roost substrate (Marshall, 1970; Dick and Patterson, 2006; Dittmar *et al*., 2015). While both species of fly are wingless and rely on their hosts for long-distance dispersal, bat flies are agile walkers and could be capable of frequent movements between individual hosts within a roost (Dick and Patterson, 2006; Dittmar *et al*., 2015). Both fly species have been documented across much of their respective hosts’ ranges (Theodor, 1957; Billeter *et al*., 2012; Qiu *et al*., 2020; Reeves *et al*., 2020; Atobatele *et al*., 2023), but no studies have evaluated their potential genetic differentiation by geography. Only a few population genetic studies have been performed on nycteribiid bat flies generally (Olival *et al*., 2013; van Schaik *et al*., 2015, 2018; Witsenburg *et al*., 2015; Speer *et al*., 2019; Verrett *et al*., 2022).

The 2 taxa of bacteria frequently associated with bat flies, *Bartonella* and *Enterobacterales*, provide an interesting contrast in their relationships with their bat and bat fly hosts. Bartonellae are associated with both bats (host) and bat flies (vector), while *Enterobacterales* symbionts are only associated with bat flies and are not hosted by bats (Dick and Dittmar, 2014; Zhu *et al*., 2014; Dittmar *et al*., 2015; Wilkinson *et al*., 2016). Bartonellae are facultative intracellular microparasites that produce long-lasting infection in host erythrocytes and are horizontally transmitted among hosts by haematophagous arthropod ectoparasites (Harms and Dehio, 2012). *Bartonella* isolates have been characterized from both *E. helvum* and *R. aegyptiacus* and similar sequences have been found in *C. greefi* and *E. africana* bat flies (Kosoy *et al*., 2010; Billeter *et al*., 2012; Kamani *et al*., 2014; Bai *et al*., 2015, 2018; Qiu *et al*., 2020; Szentiványi *et al*., 2023; Špitalská *et al*., 2024), suggesting that bat flies are vectors of these bacteria. The diversity of bartonellae infecting *E. helvum* is especially high, including at least 6 genogroups that meet criteria for recognition as distinct species (Bai *et al*., 2015). This diversity facilitates not only the potential identification of population structure in separate *Bartonella* genogroups, but also community structure in terms of the relative abundances of different *Bartonella* genogroups (Fig. 1). In comparison with *Bartonella*, the order *Enterobacterales* (including the genera *Arsenophonus* and *Aschnera*) are obligate endosymbionts of bat flies and other arthropods (Duron *et al*., 2008, 2014; Nováková *et al*., 2009; Morse *et al*., 2012, 2013; Wilkinson *et al*., 2016). They are thought to be vertically transmitted from mother to offspring *via* bacteriocytes in the milk glands of nycteribiids and may have mutualistic relationships with bat flies (Hosokawa *et al*., 2012; Dittmar *et al*., 2015). Other studies have reported these endosymbionts in *E. africana* and other *Eucampsipoda* species (Morse *et al*., 2013), and in *Cyclopodia dubia*, a congener of *C. greefi* parasitizing *Eidolon dupreanum* in Madagascar (Wilkinson *et al*., 2016). However, these studies have been limited in their geographic extent and have not attempted to identify signals of population structure in these symbionts that may reflect restrictions of bat fly dispersal.

Using this complex system involving bacteria that range from mutualistic to parasitic within their hosts, we tested the effects of geographic restrictions in host bat dispersal on microbial population or community structure across trophic levels. We hypothesize that the genetic structure of bat flies will reflect that of their specific bat hosts, with distinct haplotypes associated with mainland and island populations. Since *Enterobacterales* are obligate endosymbionts relying entirely on nycteribiid hosts for survival, we predict that these bacteria will mirror the phylogenetic separation in their bat fly host species. Similar to other vector-borne microparasite systems (Levin and Parker, 2013; Witsenburg *et al*., 2015), we expect to see no population genetic structure in the separate *Bartonella* genogroups found in flies. However, it is possible that the relative abundances of *Bartonella* genogroups detected in bat fly species will differ across sampled host populations due to host movement patterns (Fig. 1). Results from this investigation could identify evidence for the dispersal of bat flies and their symbionts through cryptic, non-reproductive movements of bats that are not captured in their genetic population structure. In addition to evaluating the differentiation of symbiont populations and communities, we assessed patterns in the prevalence of *Bartonella* bacteria across locations – particularly the influence of bat age structure and bat fly density – to better understand how these bacteria are maintained in host populations. Knowledge of bat movements across isolated islands and mainland Africa will shed light on their phylogeography, population status and conservation, and their potential to transmit other infectious agents. The results of this study will also increase our understanding of the ecological processes affecting community diversity in vector-borne parasite systems.

## Materials and methods

### Specimen collection

Bat flies were collected opportunistically during the course of a long-term research programme on the demographics, genetic population structure and viral transmission dynamics of *E. helvum* across Africa and the Gulf of Guinea islands from 2009 to 2016 (Peel *et al*., 2013, 2016, 2017; Baker *et al*., 2014). This sampling occasionally captured other fruit bat species as by-catch, including *R. aegyptiacus* on São Tomé and Príncipe. While *R. aegyptiacus* is present on Bioko (Juste and Ibáñez, 1993; Kwiecinski and Griffiths, 1999; Stribna *et al*., 2019), this species was not sampled from this island as part of this study. Additional bat capture and bat fly sampling targeting *R. aegyptiacus* in central Ghana was performed in 2012 and 2016. Permits for bat capture and sampling were granted by national and local authorities and under ethics approval from the Zoological Society of London Ethics Committee (WLE/0489 and WLE/0467); field protocols followed the American Society of Mammalogists guidelines (Sikes *et al*., 2011). Fruit bats were captured using mist nets (6–18 m; 38 mm) as bats departed roost sites at dusk or were returning at dawn. Bats were held in individual cloth bags until processing, wherein bat flies were removed from the pelage of all captured bat species while under manual restraint. Flies obtained from both species were stored in 1.2 mL microcentrifuge tubes pooled by individual bat. A minority of flies from Ghana (*n* = 18) were collected from the clothes of researchers while processing bats or on the ground under roosts (presumably groomed off and returning to the roost). The flies collected under roosts or from clothes were attributed to *E. helvum* based on the bats being sampled at the time or the predominant species in the roost and were pooled in 1.2 mL microcentrifuge tubes by date and researcher name. Pooled flies were stored either without media in a cool box before freezing or in ethanol, and then stored at 4 or −20°C until shipment. Flies were initially shipped on dry ice to the Zoological Society of London, then to the Centers for Disease Control and Prevention Division of Vector-Borne Diseases, where flies were stored at −80°C until processing. Distances between islands in the Gulf of Guinea and the mainland (considering Ghana as representative of the mainland population) were measured on Google Earth (http://earth.google.com). Age distributions of *E. helvum* populations from sampling locations were taken from Peel *et al*. (2017). Genetic data from *E. helvum*, specifically pairwise distances between populations from mitochondrial DNA (mtDNA) sequences [cytochrome b (*cytb*)] and microsatellite loci, were taken from Peel *et al*. (2013).

### Laboratory methods

Bat flies were initially identified to species based on host associations and morphological traits (Theodor, 1955, 1957, 1967). Whole bat flies were surface sterilized following published procedures (Billeter *et al*., 2012) and then homogenized in Navy Eppendorf bead tubes (Next Advance, Averill Park, NY, USA) containing 400 *μ*L of brain heart infusion (CDC, Atlanta, GA, USA) using a Bullet Blender Gold (Next Advance) until no visible appendages remained. Tubes were briefly centrifuged and a 200 *μ*L aliquot of homogenate was taken for DNA extraction. DNA was extracted from homogenates using the KingFisher Flex Purification System and associated MagMAX Pathogen RNA/DNA Kit (ThermoFisher, Waltham, MA, USA) following manufacturer protocols and then stored at 4°C during the molecular haplotyping process.

A subset of flies was haplotyped through polymerase chain reaction (PCR) amplification and sequencing of 2 mtDNA genes, 16S ribosomal RNA (rRNA) and *cytb*. These markers have previously been used for identification of species and detection of intraspecific diversity in bat flies (Dittmar *et al*., 2006; Hosokawa *et al*., 2012; Olival *et al*., 2013; Bai *et al*., 2018). *Enterobacterales* symbionts of bat flies were detected by amplification of the 16S rRNA gene (Duron *et al*., 2008). *Bartonella* DNA was amplified and sequenced at 3 markers commonly used for detection and characterization of bartonellae (La Scola *et al*., 2003; Gutiérrez *et al*., 2017; Kosoy *et al*., 2018): 16S–23S rRNA intergenic spacer region (ITS), citrate synthase gene (*gltA*) and cell division protein gene (*ftsZ*). These 3 genes are among the most frequently used markers for *Bartonella* detection and genotyping, facilitating phylogenetic comparisons with other sequences, and are able to detect low quantities of DNA in environmental samples, especially in their nested forms (Bai *et al*., 2016; Kosoy *et al*., 2018).

All PCR primers and protocols are listed with their associated references in Tables S1–S2. Preparation of PCR reagents was performed in a separate room from amplification to prevent cross-contamination. Extraction and negative (nuclease-free water) controls were used in all reactions to detect contamination of reagents. *Bartonella doshiae* was used as a positive control in all reactions for *Bartonella* detection to identify appropriately sized products. No positive controls were used for the mtDNA and *Enterobacterales* symbionts, but a DNA ladder was used to identify amplicons of approximately correct size: ~400 bp for mitochondrial 16S rRNA, ~380 bp of *cytb* and ~570 bp for bacterial 16S rRNA. Amplification products were visualized by gel electrophoresis using 1.5% agar and GelGreen stain (Biotium, Hayward, CA, USA) and then purified using a QIAquick PCR Purification Kit (QIAGEN, Valencia, CA, USA) following manufacturer's instructions. Purified products were prepared for sequencing using Big Dye terminator mix (Applied Biosystems, Inc., Foster City, CA, USA) and the same primers as PCR (the second-round primers in the case of nested *ftsZ* and *gltA* protocols) and then sequenced in both directions on an ABI 3130 Genetic Analyser (Applied Biosystems). Sequence reads were assembled with the SeqMan Pro program in Lasergene v14 (DNASTAR, Madison, WI, USA) and manually checked for ambiguous bases before phylogenetic analysis. Sequences were validated as the correct gene and target organism using the Basic Local Alignment Search Tool (BLAST; https://blast.ncbi.nlm.nih.gov/Blast.cgi).

Due to the potential amplification biases of each primer set towards different *Bartonella* genogroups in a sample, the sequences obtained from the 3 targeted genes were considered as independent measurements of the community of *Bartonella* genogroups in a sample. The presence of coexisting genogroups was confirmed in many samples through observation of multiple peaks in the electropherograms, which were separated into distinct sequences by comparison with previously obtained *Bartonella* sequences from the target bat and bat fly species (Kosoy *et al*., 2010; Billeter *et al*., 2012; Bai *et al*., 2015, 2018; McKee *et al*., 2021). Presence/absence of *Bartonella* genogroups in each bat fly was then summarized as total counts across sampling locations.

### Phylogenetic analysis

Sequences from each locus were aligned with closely matching references from GenBank using the local, iterative method L-INS-i in MAFFT v7.187 (Katoh and Standley, 2013) and trimmed to equal length with Gblocks v0.91b (Castresana, 2000). Evolutionary model selection and maximum likelihood phylogeny reconstruction for *Bartonella* sequences, haplotyped mitochondrial loci and *Enterobacterales* symbiont sequences were performed using IQ-Tree v2.1.1 (Nguyen *et al*., 2015; Minh *et al*., 2020). The top-ranking models for each set of sequences according to the Bayesian information criterion (BIC) were used for phylogenetic analysis (Schwarz, 1978; Kalyaanamoorthy *et al*., 2017). Branch support was estimated using 1000 ultrafast bootstrap replicates (Hoang *et al*., 2018). Distinct haplotypes of mitochondrial loci and *Enterobacterales* symbionts were delineated by single nucleotide changes and the observed counts of haplotypes were assessed across sampling locations. *Bartonella* sequences were assigned into separate genogroups based on phylogenetic clustering into well-supported (>70% bootstrap support) monophyletic clades with closely matching reference sequences in the maximum likelihood trees, separately for each of the 3 gene targets (*ftsZ*, *gltA* and ITS). To display how *Bartonella* genogroups are arranged within the broader phylogeny of the genus, we generated a consensus tree from concatenated *ftsZ* and *gltA* sequences. Sequences from named *Bartonella* species (including *Bartonella rousetti*; Kosoy *et al*., 2010), representative strains of genogroups E1–E5 and Ew (Bai *et al*., 2015), and sequences representing genogroups Eh6 and Eh7 from a longitudinal study on *Bartonella* in a captive colony of *E. helvum* in Ghana (McKee *et al*., 2021) were aligned for each gene, trimmed to equal length and concatenated before model selection and maximum likelihood analysis using IQ-Tree.

### Statistical analysis

*Bartonella* diversity in bat flies sampled from each location was calculated as richness, the Shannon number (the exponentiated form of Shannon entropy) and the inverse Simpson index in the R package ‘vegan’ (Oksanen *et al*., 2007; R Core Team, 2023). Confidence intervals for bat fly prevalence on bats, *Bartonella* prevalence in bat flies and *Enterobacterales* symbiont prevalence in bat flies were estimated using Wilson score intervals (Wilson, 1927). Complete metadata on bat captures was not available for all locations, so bat fly prevalence was only calculated for *E. helvum* from the Gulf of Guinea islands. The presence of *Enterobacterales* symbionts was only tested in a subset of bat flies due to inadequate sample volume following repeat testing. Since samples from each location were subdivided onto different plates for extraction, the proportion of original samples that were tested for symbionts varied across locations: 44% from Ghana, 78% from Bioko, 91% from Príncipe, 82% from São Tomé and 73% from Annobón. Two-sided *χ*^2^ tests of proportions and binomial regression models were used to test differences in bat fly prevalence across Gulf of Guinea islands, bat age class and bat sex, as well as differences in *Bartonella* prevalence across sampling locations, bat age classes and bat sex. Additional *χ*^2^ tests and binomial regression models were run on *Bartonella* prevalence across sampling years to test whether detectability of these bacteria was lower in older samples. Kruskal–Wallis rank-sum tests and Poisson regression models were used to test differences in bat fly counts on *E. helvum* across sampling locations, bat age classes and bat sex. *P* values for post-hoc comparisons from regression models were adjusted for multiple tests using the Tukey method (Tukey, 1949).

*Bartonella* community dissimilarity was calculated as 1 minus the Spearman rank correlation among *Bartonella* genogroup counts across loci between locations, aggregated across all tested bat flies. Isolation by distance patterns between islands and the mainland, as well as between each island, was explored using matrices of *Bartonella* community dissimilarity, physical distance between locations and genetic distances between bat populations (mtDNA and microsatellites) taken from Peel *et al*. (2017) using Mantel tests based on Pearson's correlation (Mantel, 1967). Additional tests were performed on *Bartonella* community composition across locations, using *Bartonella* genogroup counts within individual bat flies to calculate a Euclidean distance matrix for relative abundance. We performed univariate permutational multivariate analysis of variance (PERMANOVA) across sampling locations with 999 permutations using the *adonis2* function in ‘vegan’ (Oksanen *et al*., 2007). Homogeneity of dispersion for *Bartonella* communities across locations was tested using the *betadisper* function and permuted 999 times with *permutest* (Oksanen *et al*., 2007). Non-metric multidimensional scaling (NMDS) ordination was used to visualize differences in *Bartonella* communities between locations using the *metaMDS* function with 3 dimensions and 250 random starts to find a stable solution (Oksanen *et al*., 2007).

## Results

### Collection and identification of bat flies

Bat flies were obtained from *E. helvum* from Ghana, Bioko, Príncipe, São Tomé and Annobón, while flies from *R. aegyptiacus* were obtained only from Ghana, Príncipe and São Tomé (Table 1). A total of 767 flies were initially identified by morphology using available keys and known host distributions (Theodor, 1955, 1957, 1967). For a subset of 401 flies, sequences were successfully obtained from 1 or both 16S rRNA or *cytb* loci. All flies from *E. helvum* were identified as *C. greefi* Karsch, 1884, while flies from *R. aegyptiacus* were *E. africana* Theodor, 1955 except for a single *Dipseliopoda biannulata* Oldroyd, 1953 from Ghana (Table 2; Table S3). All 3 species are part of the Old World family Nycteribiidae, subfamily Cyclopodiinae (Maa, 1965).

**Table 1.** Sampling sites and dates for bat flies from Ghana and Gulf of Guinea islands

Bat host species	Country/island	Sampling dates	Region/site	Latitude	Longitude	Samples
*Eidolon helvum*	Ghana	25–26 March 2009; 17 January 2012; 26 March 2016–5 May 2016	Accra, 37 Hospital	5.5882	−0.1824	151
			Brong Ahafo, Tanoboase	7.6466	−1.8824	7
	Bioko	21–26 May 2010	Malabo, Hess compound	3.7471	8.7701	6
			Malabo, New Spanish embassy	3.7521	8.7723	170
	Príncipe	5–12 April 2010	Micoto	1.6802	7.3895	2
			Novo	1.5897	7.3373	79
	São Tomé	19 March–23 April 2010	Binda	0.2333	6.4833	9
			Canecao	0.3406	6.5629	7
			Cruzeiro	0.2861	6.6781	3
			Monte Cehada/Isla Calici	0.0223	6.5166	30
			Ponta Baleia	0.0430	6.5443	75
			Porto Alegre	0.0289	6.5320	41
	Annobón	10–14 May 2010	Adjo/Mábana	−1.4592	5.6453	131
*Rousettus aegyptiacus*	Ghana	25 January 2012; 7–8 May 2016	Brong Ahafo, Buoyem Cave	7.6681	−1.9617	45
	Príncipe	10–11 April 2010	Novo	1.5897	7.3373	1
	São Tomé	30 March 2010	Ponta Baleia	0.0430	6.5443	10

**Table 2.** Molecular haplotyping and *Bartonella* infection prevalence in bat flies

Bat host species	Bat fly species	Location	Samples	Haplotyped	*Bartonella* positive	Prevalence
*Eidolon helvum*	*Cyclopodia greefi*	Ghana	158	50	131	0.83 (0.76–0.88)
		Bioko	176	52	113	0.64 (0.57–0.71)
		Príncipe	81	55	67	0.83 (0.73–0.89)
		São Tomé	165	95	137	0.83 (0.77–0.88)
		Annobón	131	96	121	0.92 (0.87–0.96)
*Rousettus aegyptiacus*	*Eucampsipoda africana*	Ghana	44	41	19	0.42 (0.29–0.57)
		Príncipe	10	10	4	0.4 (0.17–0.69)
		São Tomé	1	1	1	1 (0.21–1)
	*Dipseliopoda biannulata*	Ghana	1	1	0	0 (0–0.79)

Samples were considered successfully haplotyped if 1 or both mitochondrial loci were successfully sequenced. Samples were considered positive for *Bartonella* bacteria if 1 or more genetic markers produced a sequence confirmed as *Bartonella*. Binomial 95% confidence intervals for prevalence were estimated using Wilson score intervals.

**Figure 3.**
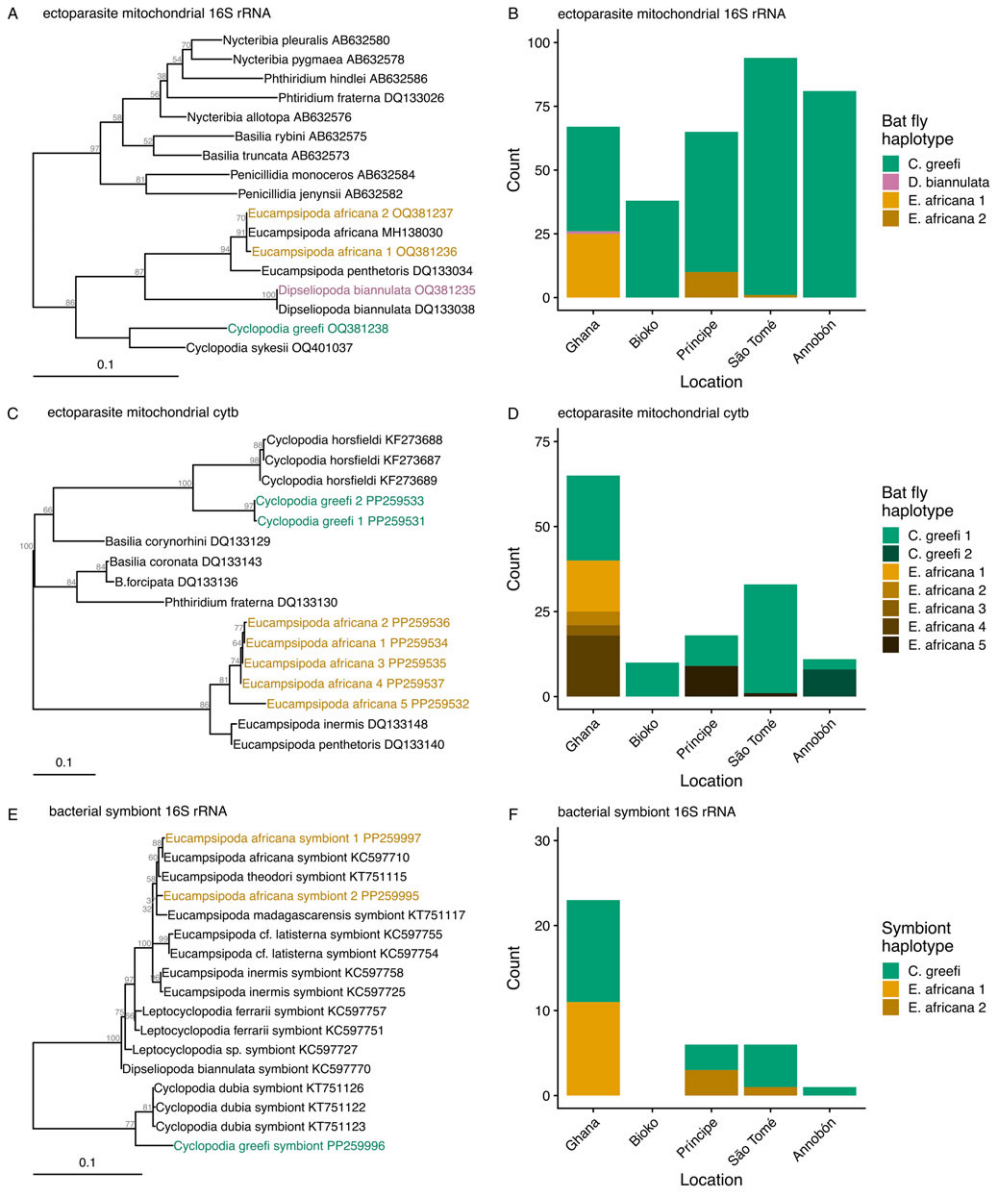
Haplotyping of bat fly species and *Enterobacterales* symbionts. Bat fly species were identified by sequencing 375 bp of mitochondrial 16S rRNA (A) and 387 bp of *cytb* (C) while bacterial symbionts of flies were identified by sequencing 575 bp of bacterial 16S rRNA (E). Maximum likelihood trees were generated in IQ-Tree using the appropriate substitution models based on BIC (TIM2 + F + G4 for ectoparasite mitochondrial 16S rRNA, TIM + F + G4 for *cytb*, K2P + R2 for bacterial symbiont 16S rRNA). Nodal support (shown in grey next to branches) was estimated from 1000 bootstrap iterations. GenBank accession numbers are given next to published reference sequences. Observed counts of haplotypes across locations (B, D and F) are shown based on the total number of specimens haplotyped at each marker. In all panels, the colours indicate separate bat fly species and symbionts: *Cyclopodia greefi* (green), *Eucampsipoda africana* (orange) and *Dipseliopoda biannulata* (pink).

The 2 mitochondrial loci revealed varying numbers of haplotypes across bat fly species (Fig. 3A–D). Only one 16S rRNA haplotype was found in *C. greefi* from all locations (Fig. 3A, B) while 2 *cytb* haplotypes were found in this species: haplotype 1 in all locations and haplotype 2 only on Annobón (Fig. 3C, D). Three individuals from Annobón were confirmed as *cytb* haplotype 1 through repeated sequencing. Two 16S rRNA haplotypes were found in *E. africana* (Fig. 3A, B). Haplotype 1 was found in Ghana and was identical to sequences from *E. africana* on GenBank (accession numbers MH138030, MH138031, MH138033–MH138037) from a previous study in Nigeria (Bai *et al*., 2018). Haplotype 2 was found in specimens from both Príncipe and São Tomé. Five *cytb* haplotypes were found in *E. africana* (Fig. 3C, D): haplotypes 1–4 were from Ghana and haplotype 5 from Príncipe and São Tomé.

Nycteribiid prevalence and the number of flies per bat varied across the different Gulf of Guinea islands and other demographic groups (Table 3). Nycteribiid prevalence differed significantly across islands (*χ*^2^ = 35, d.f. = 3, *P* < 0.001). Prevalence values on Príncipe (60%) and São Tomé (73%) were significantly lower (*P* < 0.01) than both Annobón (92%) and Bioko (91%), but differences between Príncipe and São Tomé were not significant (*P* > 0.05). Nycteribiid prevalence also differed significantly across *E. helvum* age groups (*χ*^2^ = 9.6, d.f. = 3, *P* = 0.02). Prevalence decreased across older age groups of bats: 93% in neonates, 80% in juveniles, 81% in sexually immature adults and 75% in adults. Differences in prevalence were significant only for neonates *vs* adults (*P* = 0.02) and were not significant for comparisons among other age groups. There was no significant difference in nycteribiid prevalence between sexes: 78% in female *E. helvum vs* 83% in males (*χ*^2^ = 0.9, d.f. = 1, *P* = 0.33). According to Kruskal–Wallis tests, Nycteribiid counts on infested bats did not differ significantly across locations (*χ*^2^ = 2.7, d.f. = 3, *P* = 0.45) or sexes (*χ*^2^ = 0.8, d.f. = 1, *P* = 0.36), and there were no significant post-hoc comparisons of nycteribiid counts between islands. However, Poisson regression identified significantly higher mean nycteribiid counts on males *vs* females (*P* = 0.024). Nycteribiid counts did differ across bat age classes (Kruskal–Wallis *χ*^2^ = 7.6, d.f. = 3, *P* = 0.047), averaging 3.0 (IQR 2–4) flies per bat on adults, 2.3 (IQR 1–3) on sexually immature adults, 2.5 (IQR 1–3) on juveniles and 2.3 (IQR 1–3) on neonates (Table 3). Post-hoc comparisons between age groups were not significant, though comparisons between adults *vs* sexually immature adults (*P* = 0.082) and between adults and neonates (*P* = 0.063) were borderline significant.

**Table 3.** Patterns of nycteribiid bat fly infestation prevalence on *E. helvum* sampled from the Gulf of Guinea islands

Sample group	Total bats	Bats with nycteribiids	Nycteribiid prevalence	Bats with nycteribiid count data	Nycteribiid count
Location					
Bioko	105	96	0.91 (0.85–0.95)	90	2.54 (1–3)
Príncipe	62	37	0.6 (0.47–0.71)	32	2.69 (1–3)
São Tomé	103	75	0.73 (0.64–0.8)	67	2.48 (2–3)
Annobón	75	69	0.92 (0.84–0.96)	54	3 (2–4)
Age group					
Neonate (<2 months; includes free-flying dependent young)	67	62	0.93 (0.84–0.97)	58	2.34 (1–3)
Juvenile (2–<6 months)	46	37	0.8 (0.67–0.89)	35	2.49 (1–3)
Sexually immature (6–<24 months)	70	57	0.81 (0.71–0.89)	50	2.34 (1–3)
Adult (≥24 months)	162	121	0.75 (0.67–0.81)	100	3.03 (2–4)
Sex					
Female	162	126	0.78 (0.71–0.83)	109	2.39 (2–3)
Male	183	151	0.83 (0.76–0.87)	134	2.86 (1–3.75)

Nycteribiid prevalence was calculated based on the number of bats with nycteribiids present out of the total bats captured at the location. Binomial 95% confidence intervals for prevalence were estimated using Wilson score intervals. Nycteribiid count data are displayed as the mean count on bats with nycteribiids present (excluding zeroes); the range next to the mean is the interquartile range (IQR).

### Patterns of *Bartonella* prevalence and diversity

*Bartonella* DNA was present in bat flies collected from both *E. helvum* and *R. aegyptiacus* (Table 2). On average, *Bartonella* prevalence was higher in flies collected from *E. helvum* (80%) than in flies collected from *R. aegyptiacus* (42%; *χ*^2^ = 41, d.f. = 1, *P* < 0.001). Prevalence differed across locations for *C. greefi* collected from *E. helvum* (*χ*^2^ = 42.2, d.f. = 4, *P* < 0.001), and Bioko island had significantly lower prevalence (*P* < 0.03) *vs* all other locations (Table 2). *Bartonella* prevalence did not differ across locations for *E. africana* collected from *R. aegyptiacus* (*χ*^2^ = 1.4, d.f. = 2, *P* = 0.5). We also examined differences in *Bartonella* prevalence across sampling years (Table S4). There were no significant differences in prevalence over sampling years for *C. greefi* (*χ*^2^ = 1.5, d.f. = 4, *P* = 0.68) or *E. africana* (*χ*^2^ = 1.9, d.f. = 2, *P* = 0.38). Likewise, there was no significant linear trend in prevalence over time for either species (*C. greefi*: *b* = 0.05, *z* = 1.04, *P* = 0.3; *E. africana*: *b* = 0.1, *z* = 0.89, *P* = 0.37). These findings indicate that time since sampling does not appear to be a substantial confounding factor in observed patterns of prevalence across locations.

**Figure 4.**
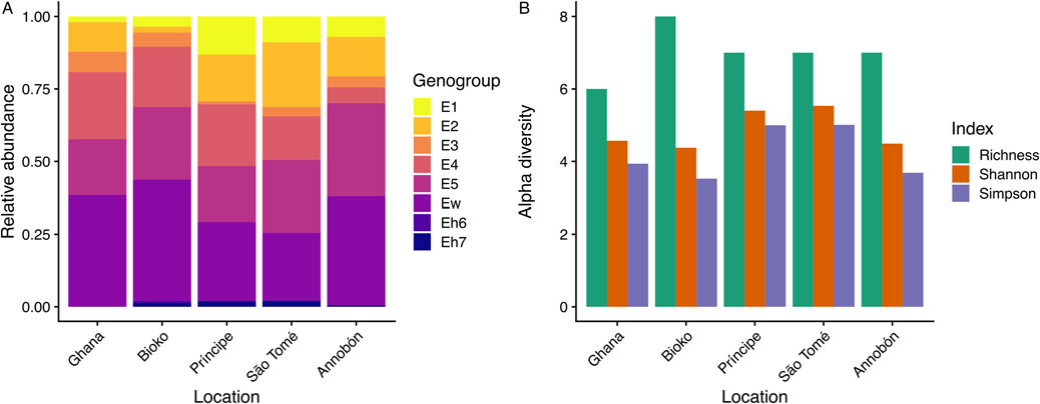
Patterns of *Bartonella* diversity in *C. greefi* bat flies collected from *E. helvum*. (A) Relative abundance of 8 *Bartonella* genogroups across sampling locations. (B) *Bartonella* genogroup alpha diversity across locations according to richness, Shannon number and inverse Simpson index.

Eight *Bartonella* genogroups were detected in *C. greefi*: E1–E5, Ew, Eh6 and Eh7 (Figs S1–S7; Fig. 4A). Genogroups E1–E5 and Ew have been detected previously in *C. greefi* and *E. helvum* from other locations and characterized at multiple genetic markers to verify their status as distinct species (Kosoy *et al*., 2010; Billeter *et al*., 2012; Kamani *et al*., 2014; Bai *et al*., 2015; McKee *et al*., 2021). In contrast, only 1 genogroup was found in *E. africana* flies from *R. aegyptiacus* (Figs S1–S7; Table S5). This genogroup, identified from cultured isolates as *B. rousetti*, has been found in *R. aegyptiacus* sampled to date from Kenya, Nigeria, Zambia, South Africa and several countries in the Middle East (Kosoy *et al*., 2010; Bai *et al*., 2018; Qiu *et al*., 2020; Szentiványi *et al*., 2023; Špitalská *et al*., 2024). To test whether genogroups Eh6 and Eh7 likely represent distinct *Bartonella* species according to the criteria established by La Scola *et al*. (2003), we compared sequences from these genogroups to the other *Bartonella* genogroups in *E. helvum* and *C. greefi* or other named *Bartonella* species. The closest match for *ftsZ* from Eh6 (MN250783) was 87% sequence identity with *Bartonella koehlerae* while the closest match for *ftsZ* from Eh7 (MN250763), *Bartonella birtlesii*, shared 88% identity. For *gltA* sequences, the closest matches were *B. koehlerae* (87%) for Eh6 (MN250780) and E4 (89%) or *Bartonella alsatica* (88%) for Eh7 (MN250763). These shared identities are below median sequence identity values for closely related *Bartonella* species (94.4% for *ftsZ* and 93.6% for *gltA*) (La Scola *et al*., 2003), suggesting that genogroups Eh6 and Eh7 are distinct species.

Unlike the situation in bat flies collected from *R. aegyptiacus*, *Bartonella* diversity varied across locations for flies collected from *E. helvum* (Table S5). *Bartonella* genogroups E1–E5 and Ew were found in *C. greefi* from Ghana and all islands whereas the rare genogroups Eh6 and Eh7 were detected inconsistently (Fig. 4A). The highest *Bartonella* richness in *C. greefi* was from Bioko whereas the highest evenness (Shannon number and inverse Simpson index) was in flies from Príncipe and São Tomé (Fig. 4B). No clear evidence of population structure was found in *Bartonella* genogroups at any of the sequenced markers (ITS, *ftsZ*, *gltA*). Identical sequences within each genogroup could be found broadly across sampling locations, including on isolated islands (Figs S1–S6).

**Figure 5.**
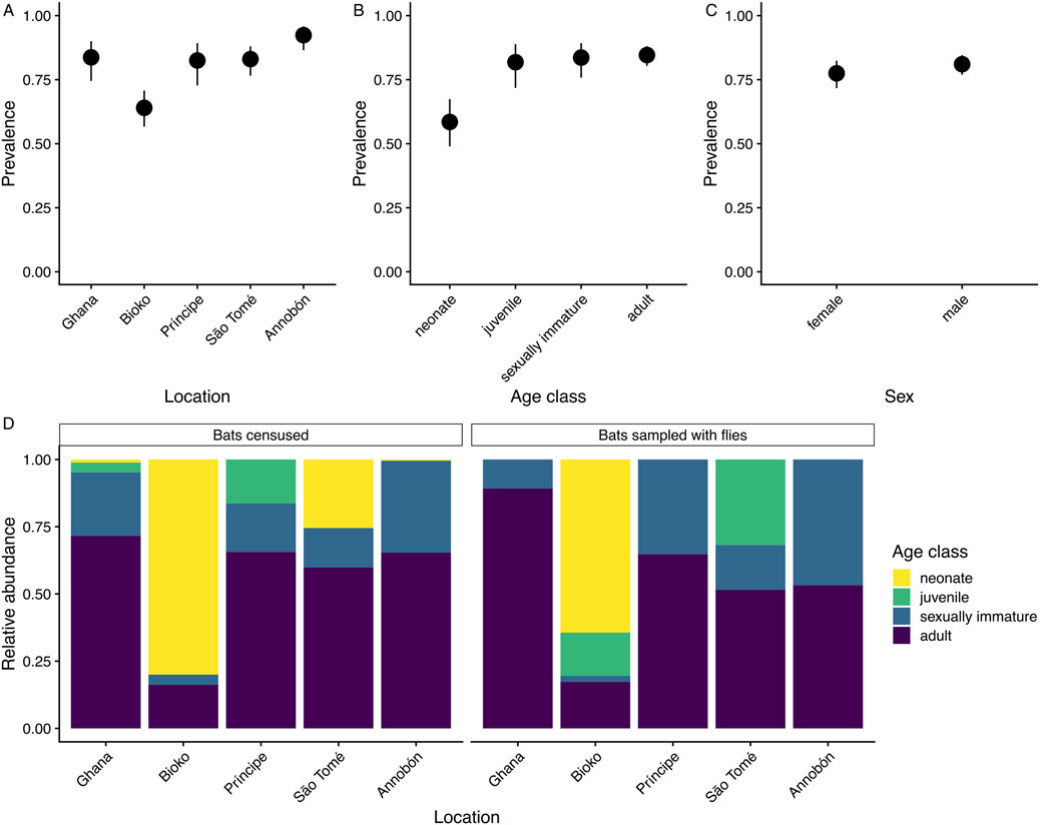
Demographic correlates of *Bartonella* detection in *C. greefi* bat flies collected from *E. helvum*. *Bartonella* detection prevalence in bat flies was calculated by (A) location, (B) bat age class and (C) bat sex and was based on the total positive bat flies collected from all bats. Binomial 95% confidence intervals for prevalence were estimated using Wilson score intervals. (D) Age distribution of *E. helvum* censused and sampled with flies from each location (and flies were tested for *Bartonella*). Note that many individuals captured on Bioko island in May 2010 were free-flying dependent young that were less than 2 months old (below the age cut-off for juveniles), so are thus lumped with other neonates.

We found that variation in *Bartonella* prevalence in *C. greefi* populations from different locations can be explained by demographic covariates (Fig. 5). *Bartonella* prevalence in *C. greefi* differed significantly across age groups of *E. helvum* that flies were collected from (*χ*^2^ = 36, d.f. = 3, *P* < 0.001). Prevalence values were significantly lower (*P* < 0.01) in neonates (58%) compared to all other age groups: juveniles (82%), sexually immature adults (84%) and adults (85%). None of the post-hoc comparisons between older age groups were significant. *Bartonella* prevalence in bat flies did not differ significantly by bat sex (77% in females *vs* 81% in males; *χ*^2^ = 0.9, d.f. = 1, *P* = 0.33; Fig. 5C). Age distributions in censused *E. helvum* populations varied widely across locations at the time of sampling (Peel *et al*., 2017; Table S6) and this was partly reflected in the representation of bat age classes among the bats with nycteribiids that were tested as part of this study (Fig. 5D). In particular, the individuals sampled on Bioko island consisted almost entirely of bats that were less than 2 months old [free-flying dependent young; termed neonates by Peel *et al*. (2017)]. This was due to inadvertent selection of a sampling site near a nursery roost and the night-time capture of bats during a time when mothers were leaving their offspring in a creche overnight (Peel *et al*., 2017). *Bartonella* prevalence was lowest in flies from Bioko (Table 2; Fig. 5A). After removing Bioko, the only location with flies sampled from neonate bats, the differences in *Bartonella* prevalence across juveniles, sexually immature adults and adults were not statistically significant (*χ*^2^ = 0.4, d.f. = 2, *P* = 0.8) and *Bartonella* prevalence no longer varied significantly across the remaining locations (*χ*^2^ = 6.7, d.f. = 3, *P* = 0.084).

**Figure 6.**
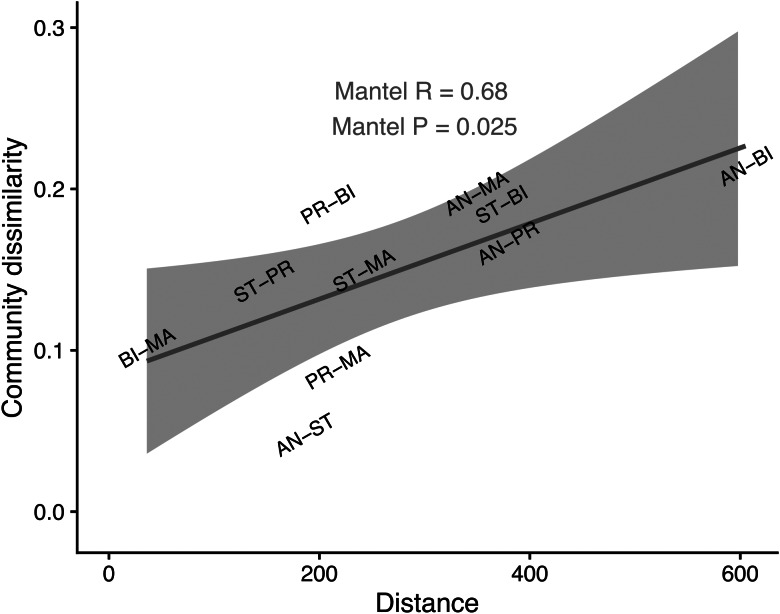
Correlation between *Bartonella* community dissimilarity in *C. greefi* and physical distance between locations. Mantel tests based on Pearson's correlation were performed with 119 permutations (the complete set for the 5 × 5 matrices). Physical distances match segments in Fig. 2B, considering Ghana as a representative mainland population. Community dissimilarity was calculated as 1 minus the Spearman rank correlation between *Bartonella* genogroup counts across locations. Locations are abbreviated as AN, Annobón; BI, Bioko; MA, mainland (Ghana); PR, Príncipe; ST, São Tomé.

To investigate the dissimilarity in *Bartonella* communities in *C. greefi* between locations, we considered the Ghanaian population to be representative of the African mainland (Peel *et al*., 2013) and we assessed the correlation between *Bartonella* community dissimilarity (based on Spearman rank correlation of aggregate *Bartonella* genogroup counts) and distance between each island and the mainland, and between each island (Figs 2B, 6). We found a positive signal of isolation by distance in *Bartonella* community dissimilarity (Mantel test *R* = 0.68, *P* = 0.025). Based on data from Peel *et al*. (2013), similar isolation by distance patterns was observed for *E. helvum* according to ϕ_ST_ [ϕ_ST_/(1–ϕ_ST_)] for *cytb* sequences (Mantel test *R* = 0.56, *P* = 0.058) and *F*_ST_ [*F*_ST_/(1–*F*_ST_)] for microsatellites (Mantel test *R* = 0.74, *P* = 0.033), though no significant associations (Mantel *P* > 0.05) were observed between either measure of bat genetic distance and *Bartonella* community dissimilarity (Fig. S9). Additional tests of *Bartonella* community composition were performed on a Euclidean distance matrix of *Bartonella* genogroup counts within individual flies. According to PERMANOVA, *Bartonella* community composition was structured by sampling location (*R* = 0.2, *F* = 5.7, d.f. = 4, *P* = 0.001). However, we note that these data violate PERMANOVA's assumption of homoscedasticity (*F* = 3.9, d.f. = 4, *P* = 0.004). NMDS ordination showed substantial overlap in *Bartonella* community composition (Fig. S8), though Annobón diverged from the other locations, particularly in lower abundance of genogroup E4. There was also a statistically significant association between *Bartonella* community dissimilarity and physical distances between sampling locations (Mantel test *R* = 0.02, *P* = 0.004).

### Detection and identification of bat fly symbionts

*Enterobacterales* symbionts (Gammaproteobacteria) were successfully detected in bat flies from mainland and island populations (Table S8). Symbionts were challenging to detect with the 16S rRNA PCR protocol, with 4% (21/512) of *C. greefi* and 63% (15/24) of *E. africana* producing positive *Enterobacterales* results in the subset of samples that were tested. The bacteria detected in *C. greefi* were most closely related to endosymbionts from the congener *C. dubia* collected from *E. dupreanum* from Madagascar (Wilkinson *et al*., 2016). The phylogenetic group that contains the symbionts from *Cyclopodia* is distinct from other known genera of bat fly symbionts, including *Arsenophonus*, *Arsenophonus*-like organisms and *Aschnera* (Wilkinson *et al*., 2016). Only 1 haplotype of the *C. greefi* symbiont was obtained from flies collected from Ghana, Príncipe, São Tomé and Annobón (Fig. 3E, F; Table S3). The bacteria from *E. africana* were most closely related to endosymbionts detected in *Eucampsipoda*, *Leptocyclopodia* and *Dipseliopoda* spp. bat flies from bats in Kenya, China, Philippines, Madagascar and Comoros; this phylogenetic group is considered part of the genus *Arsenophonus* (Morse *et al*., 2013; Wilkinson *et al*., 2016). Two haplotypes of *E. africana* symbionts were obtained from the samples (Fig. 3E, F; Table S3). Haplotype 1 was found in flies collected from Ghana and was most closely related to a symbiont previously detected in *E. africana* from Kenya (Morse *et al*., 2013). Haplotype 2 was found in flies collected from Príncipe and São Tomé and formed a separate branch from symbionts in *E. africana* from the mainland and *E. theodori* from Comoros (Wilkinson *et al*., 2016).

## Discussion

Host–vector–microbe systems are ubiquitous, but our knowledge of the effects of host movement on the population genetics and community assembly of ectoparasites and microbes is still incomplete. Through joint analysis of ectoparasitic vectors and bacterial microparasites and symbionts, this study aimed to infer patterns of host movement beyond those reflected in population genetic analysis of hosts alone. This study contributes to our understanding of the phylogeography of African bats and nycteribiid bat flies and supports general expectations of limited genetic differentiation in vector-borne microparasites.

Sequencing mitochondrial loci from *C. greefi* and *E. africana* bat flies revealed limited population structure in both species compared with their bat hosts. A unique haplotype of *C. greefi* was found only on Annobón, which corresponds with the presence of a genetically distinct subspecies of *E. helvum* on this island (Juste *et al*., 2000; Peel *et al*., 2013). The remaining *C. greefi* specimens from Ghana, Bioko, Príncipe and São Tomé are a single haplotype, failing to capture the genetic differentiation between Príncipe and São Tomé or the distinction of these island populations from the mainland and Bioko as seen in *E. helvum* (Peel *et al*., 2013). Three individuals from Annobón had this widespread haplotype, suggesting that they represent recent immigrants to Annobón. Such distant dispersal events have been reported in *E. helvum*, including 1 individual recorded from the Cape Verde islands 570 km from the African mainland (Jiménez and Hazevoet, 2010) and another recorded travelling 370 km from its roost in Zambia in 1 night during migration (Richter and Cumming, 2008). The population structure of *E. africana* also partially mirrored that of its host, *R. aegyptiacus*. The single haplotype from Príncipe and São Tomé was distinct from the other haplotypes found on the mainland. This reflects the distinctiveness of the *R. aegyptiacus* populations from these islands compared to the mainland, but fails to distinguish the island populations from one another (Juste and Ibáñez, 1993; Stribna *et al*., 2019). These results agree with past studies that have shown less structure in bat flies compared to their hosts due to recent or ongoing gene flow (Witsenburg *et al*., 2015; van Schaik *et al*., 2018). We conclude that occasional, non-reproductive movements of *E. helvum* and *R. aegyptiacus* between islands and the mainland may contribute to the dispersal of their ectoparasitic bat flies.

A limitation of this study is that the amount of population structure seen in the flies is sensitive to the choice of genetic marker used for haplotyping. In both fly species, mitochondrial *cytb* was able to find more distinct haplotypes with greater pairwise distances than 16S rRNA. Previous studies on *E. helvum* and *R. aegyptiacus* used *cytb* to identify population structure. Peel *et al*. (2013) were able to detect differentiation in *E. helvum* populations on São Tomé and Príncipe from one another using *cytb* but not with microsatellites and Stribna *et al*. (2019) were able to distinguish São Tomé and Príncipe populations with both *cytb* and microsatellites. The 16S rRNA gene may be too conserved for this type of analysis. We suggest using other mitochondrial or nuclear loci for genotyping nycteribiid flies and ideally matching markers between hosts and ectoparasites so that mutation rates and inheritance patterns are similar.

Other factors may have affected the amount of genetic population structure observed in bat flies compared to bats, including differences in generation length, effective population size and selection pressure. Reproduction in *E. helvum* and *R. aegyptiacus* is seasonal and females give birth to 1–2 pups per year after reaching sexual maturity after 1 year in *R. aegyptiacus* (Mutere, 1968; Nkoana *et al*., 2023) and 2 years in *E. helvum* (Peel *et al*., 2016). Maximum lifespans of *E. helvum* have been recorded up to 15 years in the wild (Hayman *et al*., 2012; Peel *et al*., 2016) and 21 years in captivity (DeFrees and Wilson, 1988), while the maximum age for captive *R. aegyptiacus* is reported as 25 years (Kwiecinski and Griffiths, 1999). Although specific data on *C. greefi* and *E. africana* reproduction are not available, studies of other species living in tropical areas indicate that nycteribiid lifespans are on the order of several hundred days (Marshall, 1970, 1971). Given that development for pupal stage to adult takes about 3 weeks, this means that 70–80% of their lifespan is spent as a reproductively mature adult (Marshall, 1970, 1971). These data suggest that multiple generations of nycteribiids may be produced each year, which may have consequences for mutational fixation rates in bats *vs* bat flies. *Eidolon helvum* and *R. aegyptiacus* are abundant across their ranges, with some colonies numbering in the thousands to millions of individuals (Kwiecinski and Griffiths, 1999; Peel *et al*., 2017; Hurme *et al*., 2022). While our data (Table 3) and other published work (Atobatele *et al*., 2023) suggest that nycteribiid prevalence on *E. helvum* is high across sampled populations, most bats only carry 1–3 bat flies, suggesting that population sizes of nycteribiids are not orders of magnitude higher than that of bat hosts. Published data on *Rousettus* bats and *Eucampsipoda* bat flies indicate similar patterns (Rajemison *et al*., 2017; Pawęska *et al*., 2021). It is also possible that bat flies may be experiencing weaker selection pressure than bats because the environment they experience as parasites is relatively stable compared to the environment that bats experience. However, since nycteribiids live in the pelage of bats and must leave their hosts to pupate onto a substrate, they would experience seasonal changes in temperature, humidity and precipitation and other environmental factors that may affect their survival and reproduction. Bat flies also experience predation by their bat hosts (Overal, 1980; Ramanantsalama *et al*., 2018). Thus, while the selective pressures on bat flies may differ from host bats, these differences may not be as substantial as those experienced by endoparasites or microparasites, whose environment is inside the host's body.

The low genetic diversity observed in bat flies could also be linked to the presence of *Enterobacterales* symbionts. Previous studies have attributed the lack of population differentiation in mtDNA to selective sweeps caused by reproductive manipulation in those flies not carrying the bacterial symbiont (Hurst and Jiggins, 2005; Lack *et al*., 2011; Speer *et al*., 2019). This selection may include killing of male embryos, changing embryos from male to female or sterilization of uninfected females by infected males, all of which can decrease mtDNA diversity while selecting for mtDNA haplotypes associated with the originally infected females. While reproductive manipulation is well-documented for *Wolbachia* symbionts of insects (Cariou *et al*., 2017), it is unknown to what degree, if any, this features in the relationships between *Arsenophonus* bacteria or other *Enterobacterales* symbionts and bat flies (Duron *et al*., 2008; Lack *et al*., 2011). Sequencing of these fly populations at nuclear loci could identify additional population structure in these species and more accurately estimate the amount of gene flow occurring due to bat dispersal. Such data could also clarify the effect that *Enterobacterales* symbionts have on mitochondrial diversity.

Despite the possible interaction between bacterial symbionts and mtDNA, the population structure of *Enterobacterales* symbionts reflected the inferred dispersal patterns of their host bat flies. This fits well with expectations that vertically transmitted parasites are good proxies for inferring movement of their hosts (Nieberding and Olivieri, 2007). The unique phylogenetic group of *Enterobacterales* symbionts of *C. greefi* was genetically homogeneous across Ghana, Príncipe, São Tomé and Annobón. The presence of only 1 haplotype may reflect the occasional, indirect dispersal (via bat hosts) of bat flies carrying these bacteria between islands. The *Arsenophonus* symbionts of *E. africana* were split into 2 haplotypes that corresponded to the geographic distribution of the hosts, with 1 haplotype from Príncipe and São Tomé and the other from Ghana. As with haplotyping bat flies, bacterial 16S rRNA may be too conserved to successfully identify phylogenetically distinct haplotypes of *Enterobacterales* symbionts, and additional genes should be sequenced. These data would be useful in comparing with the diversity at nuclear loci in bat flies to better detect signatures of selective sweeps in mtDNA due to reproductive manipulation.

The patterns observed in *Bartonella* bacteria reflect their lifestyle as horizontally transmitted, vector-borne microparasites. As expected, no population genetic structure was seen in the separate *Bartonella* genogroups from *C. greefi* and *E. africana*. A previous study using multi-locus sequence typing to characterize *Bartonella* cultures from genogroups E1–E5 and Ew from *E. helvum* from African populations also found identical multi-locus sequence types that were found in geographically distant locations on the continent and from Annobón (Bai *et al*., 2015). These results are similar to previous studies that have found little correlation between the genetic structure observed in vector-borne microparasites compared to their hosts or vectors (Levin and Parker, 2013; Witsenburg *et al*., 2015) and lend support to the hypothesis that host and vector movement have additive effects on gene flow in associated microparasites (Witsenburg *et al*., 2015). While the markers used for *Bartonella* detection are sufficiently diverse to identify different *Bartonella* genogroups and species (La Scola *et al*., 2003; Kosoy *et al*., 2018), their substitution rates may still be too low to detect microevolutionary patterns. Additional studies using culturing and more extensive methods for haplotyping, such as amplified fragment-length polymorphisms or whole genome sequencing, could find additional structure. Nevertheless, by analysing the relative abundance of the diverse *Bartonella* genogroups found in *C. greefi* from *E. helvum*, a significant pattern of isolation by distance was observed, with locations nearer to each other having more similar rank abundances of genogroups, such as Ghana and Bioko or Príncipe and São Tomé. A similar pattern of isolation by distance was seen in *E. helvum* using mtDNA and microsatellites, but there was no correlation between these genetic distances and *Bartonella* community structure. Thus, it is likely that movement of bats (with their bat flies) is restricted by the distances between islands, and this results in changes in transmission patterns that affect *Bartonella* communities. We encourage future studies to consider analysing microparasite and symbiont communities as we have done, since they may help to further clarify patterns of host movements that are uncorrelated with reproduction but lead to dispersal of ectoparasites and microbes.

*Bartonella* diversity in *C. greefi* did not vary much, with the same common genogroups occurring across locations and only differing in their relative abundances (Fig. 4). This is counterintuitive given expectations of island biogeography, which would predict a lower diversity of bacterial communities on the smallest and most isolated islands. This might be explained by chronic or recurrent latent infections, continuous transmission of *Bartonella* in bats within a population, and possible transmission events between populations through occasionally dispersing bats (and bat flies). These factors could sustain populations of bartonellae and prevent the local extinctions that are a fundamental to island biogeography theory.

The *Bartonella* prevalence in both bat fly species was comparable to previous studies using similar molecular detection methods (Table 2). Billeter *et al*. (2012) reported *Bartonella* prevalence of 57% (26/46), 72% (23/31) and 71% (42/59) in *C. greefi* flies collected from *E. helvum* from Ghana, Annobón and Bioko, respectively. Bai *et al*. (2018) reported *Bartonella* prevalence of 42% (21/50) in *E. africana* flies from *R. aegyptiacus* from Nigeria. Qiu *et al*. (2020) reported prevalence of 47% (9/19) in *E. africana* flies collected in Zambia. There was no overlap in the genogroups of *Bartonella* found in *C. greefi* and *E. africana*, which reflects the specificity of these bacteria to their bat hosts (Kosoy *et al*., 2010; Bai *et al*., 2015; Qiu *et al*., 2020). This is reinforced by the ecological separation of the 2 hosts and bat fly vectors. While these bat species may interact occasionally at feeding sites, they exhibit different roosting behaviour, with *E. helvum* roosting predominantly in trees and *R. aegyptiacus* in caves. While *C. greefi* has been occasionally collected from *R. aegyptiacus* and *E. africana* from *E. helvum* (Theodor, 1955; Atama, 2015; Nartey, 2015), these infrequent exchanges of flies do not appear to lead to *Bartonella* transmission from bat flies to an atypical host.

A secondary goal of this study was to find population-level predictors of *Bartonella* prevalence across sampled populations. *Bartonella* prevalence in bat flies was related to the age of bats, but this was only observed due to inadvertent sampling of very young bats on Bioko. This agrees with results from a captive colony of *E. helvum* in Ghana, wherein neonate bats were found to be initially uninfected with *Bartonella* and became infected when bat flies were present (McKee *et al*., 2021). It is important to note that sampling periods from this study were not all contemporaneous and density of flies in a population can also vary seasonally (Atobatele *et al*., 2023) and potentially interannually, which can have implications for *Bartonella* transmission. A longitudinal study of *Bartonella* infection in bats and bat flies from Bangladesh found that *Bartonella* prevalence in bats increased over the 9-month study period, which coincided with the rainy season, an influx of juvenile bats into the population, and an increase in the prevalence of bat flies (Fagre *et al*., 2023). The study by Fagre *et al*. (2023) provides support for the hypothesis that bats become exposed to *Bartonella* relatively early in life following colonization by bat flies. We suggest that more longitudinal studies of *Bartonella* infection in bats and bat flies be performed to understand how *Bartonella* and bat fly prevalence vary seasonally and over a bat's lifespan to further understand the transmission dynamics of this microparasite.

In summary, the joint analysis of parasites and symbionts from African fruit bats has demonstrated that these organisms can reveal movement patterns and interactions among bat populations that are not apparent from analysis of host bats alone. Such movements could contribute to the maintenance of other infectious agents in these bats, including viruses (Peel *et al*., 2012; Glennon *et al*., 2019). While direct interactions with bats are generally uncommon, close contact can occur in some subpopulations that participate in bat hunting and the consumption of bat meat (Mickleburgh *et al*., 2009; Kamins *et al*., 2011; Peel *et al*., 2017; Baudel *et al*., 2019) or tourism and other cultural practices in bat caves (Fujita *et al*., 2009; Timen *et al*., 2009; Ohemeng *et al*., 2017; Vora *et al*., 2020). An understanding of the infection cycles of viruses and other bat-borne pathogens is critical for assessing the risk of spillover into human populations through various exposure routes (Pernet *et al*., 2014; Mannerings *et al*., 2016; Bai *et al*., 2018; Mbu'u *et al*., 2019; Vora *et al*., 2020). On a broader level, this study increases our knowledge of the complex ecology and population genetics of host–microbe systems that are widespread in nature.

## Supporting information

McKee et al. supplementary material 1 McKee et al. supplementary material

McKee et al. supplementary material 2 McKee et al. supplementary material

McKee et al. supplementary material 3 McKee et al. supplementary material

McKee et al. supplementary material 4 McKee et al. supplementary material

McKee et al. supplementary material 5 McKee et al. supplementary material

McKee et al. supplementary material 6 McKee et al. supplementary material

McKee et al. supplementary material 7 McKee et al. supplementary material

McKee et al. supplementary material 8 McKee et al. supplementary material

McKee et al. supplementary material 9 McKee et al. supplementary material

McKee et al. supplementary material 10 McKee et al. supplementary material

## Data Availability

The data that support the findings of this study are available in the supplementary material of this article. Representative sequences for bat fly mitochondrial haplotypes and *Enterobacterales* symbiont haplotypes are available on GenBank (OQ381235–OQ381238, PP259531–PP259537 and PP259995–PP259997). Phylogenetic trees, R code and additional data sheets are available on GitHub (https://github.com/clifmckee/GoG_bats_bat_flies).

## Supplementary material

The supplementary material for this article can be found at https://doi.org/10.1017/S0031182024000660.

## References

[ref1] Atama N (2015) Haemoparasites and ectoparasites of fruit bat species in Amurum Forest Reserve and their effects on hosts physiologic and morphometric parameters (MSc thesis), University of Jos, Jos, Nigeria.

[ref2] Atobatele OE, Olatubi IV, Oyeku OG, Ayokunle DI, Oladosu OO and Ogunnaike TM (2023) Analysis of COI gene, prevalence, and intensity of the bat fly *Cyclopodia greeffi* on roosting straw-coloured fruit bat *Eidolon helvum* in Southwest Nigeria. International Journal for Parasitology: Parasites and Wildlife 21, 210–218. 37388298 10.1016/j.ijppaw.2023.06.003 PMC10300209

[ref3] Bai Y, Hayman DTS, McKee CD and Kosoy MY (2015) Classification of *Bartonella* strains associated with straw-colored fruit bats (*Eidolon helvum*) across Africa using a multi-locus sequence typing platform. PLoS Neglected Tropical Diseases 9, e0003478. 25635826 10.1371/journal.pntd.0003478 PMC4311972

[ref4] Bai Y, Gilbert A, Fox K, Osikowicz L and Kosoy M (2016) *Bartonella rochalimae* and *B. vinsonii* subsp. *berkhoffii* in wild carnivores from Colorado, USA. Journal of Wildlife Diseases 52, 844–849. 27529290 10.7589/2016-01-015 PMC7075493

[ref5] Baker KS, Suu-Ire R, Barr J, Hayman DTS, Broder CC, Horton DL, Durrant C, Murcia PR, Cunningham AA and Wood JLN (2014) Viral antibody dynamics in a chiropteran host. Journal of Animal Ecology 83, 415–428. 24111634 10.1111/1365-2656.12153 PMC4413793

[ref6] Baudel H, De Nys H, Mpoudi Ngole E, Peeters M and Desclaux A (2019) Understanding Ebola virus and other zoonotic transmission risks through human–bat contacts: exploratory study on knowledge, attitudes and practices in southern Cameroon. Zoonoses and Public Health 66, 288–295. 30677236 10.1111/zph.12563 PMC7165775

[ref7] Biek R, Drummond AJ and Poss M (2006) A virus reveals population structure and recent demographic history of its carnivore host. Science 311, 538–541. 16439664 10.1126/science.1121360

[ref8] Billeter SA, Hayman DTS, Peel AJ, Baker K, Wood JLN, Cunningham A, Suu-Ire R, Dittmar K and Kosoy MY (2012) *Bartonella* species in bat flies (Diptera: Nycteribiidae) from western Africa. Parasitology 139, 324–329. 22309510 10.1017/S0031182011002113

[ref9] Cariou M, Duret L and Charlat S (2017) The global impact of *Wolbachia* on mitochondrial diversity and evolution. Journal of Evolutionary Biology 30, 2204–2210. 28977708 10.1111/jeb.13186

[ref10] Castresana J (2000) Selection of conserved blocks from multiple alignments for their use in phylogenetic analysis. Molecular Biology and Evolution 17, 540–552. 10742046 10.1093/oxfordjournals.molbev.a026334

[ref11] Criscione CD, Cooper B and Blouin MS (2006) Parasite genotypes identify source populations of migratory fish more accurately than fish genotypes. Ecology 87, 823–828. 16676525 10.1890/0012-9658(2006)87[823:pgispo]2.0.co;2

[ref12] DeFrees SL and Wilson DE (1988) Eidolon helvum. Mammalian Species 312, 1–5. doi: 10.2307/3504095

[ref13] Dick CW and Dittmar K (2014) Parasitic bat flies (Diptera: Streblidae and Nycteribiidae): host specificity and potential as vectors. In Klimpel S and Mehlhorn H (eds), Bats (Chiroptera) as Vectors of Diseases and Parasites: Facts and Myths. Berlin, Heidelberg: Springer, pp. 131–155. doi: 10.1007/978-3-642-39333-4_6

[ref14] Dick CW and Patterson BD (2006) Bat flies: obligate ectoparasites of bats. In Morand S, Krasnov BR and Poulin R (eds). Micromammals and Macroparasites: From Evolutionary Ecology to Management. Tokyo, Japan: Springer, pp. 179–194.

[ref15] Dittmar K, Porter ML, Murray S and Whiting MF (2006) Molecular phylogenetic analysis of nycteribiid and streblid bat flies (Diptera: Brachycera, Calyptratae): implications for host associations and phylogeographic origins. Molecular Phylogenetics and Evolution 38, 155–170. 16087354 10.1016/j.ympev.2005.06.008

[ref16] Dittmar K, Morse SF, Dick CW and Patterson BD (2015) Bat fly evolution from the Eocene to the present (Hippoboscoidea, Streblidae and Nycteribiidae. In Morand S, Krasnov BR and Littlewood TJ (eds). Parasite Diversity and Diversification: Evolutionary Ecology Meets Phylogenetics. Cambridge: Cambridge University Press, pp. 246–264.

[ref17] Duron O, Bouchon D, Boutin S, Bellamy L, Zhou L, Engelstädter J and Hurst GD (2008) The diversity of reproductive parasites among arthropods: *Wolbachia* do not walk alone. BMC Biology 6, 27. 18577218 10.1186/1741-7007-6-27 PMC2492848

[ref18] Duron O, Schneppat UE, Berthomieu A, Goodman SM, Droz B, Paupy C, Obame Nkoghe J, Rahola N and Tortosa P (2014) Origin, acquisition and diversification of heritable bacterial endosymbionts in louse flies and bat flies. Molecular Ecology 23, 2105–2117. 24612422 10.1111/mec.12704

[ref19] Fagre AC, Islam A, Reeves WK, Kading RC, Plowright RK, Gurley ES and McKee CD (2023) *Bartonella* infection in fruit bats and bat flies, Bangladesh. Microbial Ecology 86, 2910–2922. 37656196 10.1007/s00248-023-02293-9

[ref20] Falush D, Wirth T, Linz B, Pritchard JK, Stephens M, Kidd M, Blaser MJ, Graham DY, Vacher S, Perez-Perez GI, Yamaoka Y, Mégraud F, Otto K, Reichard U, Katzowitsch E, Wang X, Achtman M and Suerbaum S (2003) Traces of human migrations in *Helicobacter pylori* populations. Science 299, 1582–1585. 12624269 10.1126/science.1080857

[ref21] Fujita N, Miller A, Miller G, Gershman K, Gallagher N, Marano N, Hale C and Jentes E (2009) Imported case of Marburg hemorrhagic fever – Colorado, 2008. Morbidity and Mortality Weekly Report 58, 1377–1381. 20019654

[ref22] Glennon EE, Becker DJ, Peel AJ, Garnier R, Suu-Ire RD, Gibson L, Hayman DTS, Wood JLN, Cunningham AA, Plowright RK and Restif O (2019) What is stirring in the reservoir? Modelling mechanisms of henipavirus circulation in fruit bat hosts. Philosophical Transactions of the Royal Society B: Biological Sciences 374, 20190021. 10.1098/rstb.2019.0021 PMC6711305 31401962

[ref23] Gutiérrez R, Vayssier-Taussat M, Buffet J-P and Harrus S (2017) Guidelines for the isolation, molecular detection, and characterization of *Bartonella* species. Vector-Borne and Zoonotic Diseases 17, 42–50. 28055575 10.1089/vbz.2016.1956

[ref24] Harms A and Dehio C (2012) Intruders below the radar: molecular pathogenesis of *Bartonella* spp. Clinical Microbiology Reviews 25, 42–78. 22232371 10.1128/CMR.05009-11 PMC3255967

[ref25] Hayman DTS, McCrea R, Restif O, Suu-Ire R, Fooks AR, Wood JLN, Cunningham AA and Rowcliffe JM (2012) Demography of straw-colored fruit bats in Ghana. Journal of Mammalogy 93, 1393–1404. 23525358 10.1644/11-MAMM-A-270.1 PMC3605799

[ref26] Hoang DT, Chernomor O, von Haeseler A, Minh BQ and Vinh LS (2018) UFBoot2: improving the ultrafast bootstrap approximation. Molecular Biology and Evolution 35, 518–522. 29077904 10.1093/molbev/msx281 PMC5850222

[ref27] Holmes EC (2004) The phylogeography of human viruses. Molecular Ecology 13, 745–756. 15012753 10.1046/j.1365-294x.2003.02051.x

[ref28] Hosokawa T, Nikoh N, Koga R, Satô M, Tanahashi M, Meng X-Y and Fukatsu T (2012) Reductive genome evolution, host–symbiont co-speciation and uterine transmission of endosymbiotic bacteria in bat flies. The ISME Journal 6, 577–587. 21938025 10.1038/ismej.2011.125 PMC3280136

[ref29] Hurme E, Fahr J, Network EM, Eric-Moise BF, Hash CT, O'Mara MT, Richter H, Tanshi I, Webala PW, Weber N, Wikelski M and Dechmann DKN (2022) Fruit bat migration matches green wave in seasonal landscapes. Functional Ecology 36, 2043–2055.

[ref30] Hurst GDD and Jiggins FM (2005) Problems with mitochondrial DNA as a marker in population, phylogeographic and phylogenetic studies: the effects of inherited symbionts. Proceedings of the Royal Society B: Biological Sciences 272, 1525–1534. 10.1098/rspb.2005.3056 PMC1559843 16048766

[ref31] Bai Y, Osinubi MOV, Osikowicz L, McKee C, Vora NM, Rizzo MR, Recuenco S, Davis L, Niezgoda M, Ehimiyein AM, Kia GSN, Oyemakinde A, Adeniyi OS, Gbadegesin YH, Saliman OA, Ogunniyi A, Ogunkoya AB, Kosoy MY and Idanre Bat Festival Investigation Team (2018) Human exposure to novel *Bartonella* species from contact with fruit bats. Emerging Infectious Diseases 24, 2317–2323. 30457529 10.3201/eid2412.181204 PMC6256376

[ref32] Jarne P and Théron A (2001) Genetic structure in natural populations of flukes and snails: a practical approach and review. Parasitology 123, 27–40. 10.1017/s0031182001007715 11769289

[ref33] Jiménez S and Hazevoet CJ (2010) First record of straw-coloured fruit bat *Eidolon helvum* (Kerr, 1792) for the Cape Verde Islands. Zoologia Caboverdiana 1, 116–118.

[ref34] Juste J and Ibáñez C (1993) Geographic variation and taxonomy of *Rousettus aegyptiacus* (Mammalia: Megachiroptera) in the islands of the Gulf of Guinea. Zoological Journal of the Linnean Society 107, 117–129.

[ref35] Juste J, Machordom A and Ibañez C (1996) Allozyme variation of the Egyptian rousette (*Rousettus egyptiacus*; Chiroptera Pteropodidae) in the Gulf of Guinea (West-Central Africa). Biochemical Systematics and Ecology 24, 499–508.

[ref36] Juste J, Ibáñez C and Machordom A (2000) Morphological and allozyme variation of *Eidolon helvum* (Mammalia: Megachiroptera) in the islands of the Gulf of Guinea. Biological Journal of the Linnean Society 71, 359–378.

[ref37] Kalyaanamoorthy S, Minh BQ, Wong TKF, von Haeseler A and Jermiin LS (2017) ModelFinder: fast model selection for accurate phylogenetic estimates. Nature Methods 14, 587–589. 28481363 10.1038/nmeth.4285 PMC5453245

[ref38] Kamani J, Baneth G, Mitchell M, Mumcuoglu KY, Gutiérrez R and Harrus S (2014) *Bartonella* species in bats (Chiroptera) and bat flies (Nycteribiidae) from Nigeria, West Africa. Vector-Borne and Zoonotic Diseases 14, 625–632. 25229701 10.1089/vbz.2013.1541 PMC4170809

[ref39] Kamins AO, Restif O, Ntiamoa-Baidu Y, Suu-Ire R, Hayman DTS, Cunningham AA, Wood JLN and Rowcliffe JM (2011) Uncovering the fruit bat bushmeat commodity chain and the true extent of fruit bat hunting in Ghana, West Africa. Biological Conservation 144, 3000–3008. 22514356 10.1016/j.biocon.2011.09.003 PMC3323830

[ref40] Katoh K and Standley DM (2013) MAFFT multiple sequence alignment software version 7: improvements in performance and usability. Molecular Biology and Evolution 30, 772–780. 23329690 10.1093/molbev/mst010 PMC3603318

[ref41] Kosoy M, Bai Y, Lynch T, Kuzmin IV, Niezgoda M, Franka R, Agwanda B, Breiman RF and Rupprecht CE (2010) *Bartonella* spp. in bats, Kenya. Emerging Infectious Diseases 16, 1875–1881. 21122216 10.3201/eid1612.100601 PMC3294596

[ref42] Kosoy M, McKee C, Albayrak L and Fofanov Y (2018) Genotyping of *Bartonella* bacteria and their animal hosts: current status and perspectives. Parasitology 145, 543–562. 28764816 10.1017/S0031182017001263

[ref43] Kwiecinski GG and Griffiths TA (1999) Rousettus egyptiacus. Mammalian Species 611, 1–9. doi: 10.2307/3504411

[ref44] Lack JB, Nichols RD, Wilson GM and Van Den Bussche RA (2011) Genetic signature of reproductive manipulation in the phylogeography of the bat fly, *Trichobius major*. Journal of Heredity 102, 705–718. 21890840 10.1093/jhered/esr090

[ref45] La Scola B, Zeaiter Z, Khamis A and Raoult D (2003) Gene-sequence-based criteria for species definition in bacteriology: the *Bartonella* paradigm. Trends in Microbiology 11, 318–321. 12875815 10.1016/s0966-842x(03)00143-4

[ref46] Lee JS, Ruell EW, Boydston EE, Lyren LM, Alonso RS, Troyer JL, Crooks KR and VandeWoude S (2012) Gene flow and pathogen transmission among bobcats (*Lynx rufus*) in a fragmented urban landscape. Molecular Ecology 21, 1617–1631. 22335296 10.1111/j.1365-294X.2012.05493.x

[ref47] Levin II and Parker PG (2013) Comparative host–parasite population genetic structures: obligate fly ectoparasites on Galapagos seabirds. Parasitology 140, 1061–1069. 23659306 10.1017/S0031182013000437

[ref48] Maa TC (1965) An interim world list of batflies: (Diptera: Nycteribiidae and Streblidae). Journal of Medical Entomology 1, 377–386. 14280491 10.1093/jmedent/1.4.377

[ref49] Mannerings AO, Osikowicz LM, Restif O, Nyarko E, Suu-Ire R, Cunningham AA, Wood JLN and Kosoy MY (2016) Exposure to bat-associated *Bartonella* spp. among humans and other animals, Ghana. Emerging Infectious Diseases 22, 922–924. 27088812 10.3201/eid2205.151908 PMC4861528

[ref50] Mantel N (1967) The detection of disease clustering and a generalized regression approach. Cancer Research 27, 209–220. 6018555

[ref51] Marshall AG (1970) The life cycle of *Basilia hispida* Theodor 1967 (Diptera: Nycteribiidae) in Malaysia. Parasitology 61, 1–18.

[ref52] Marshall AG (1971) The ecology of *Basilia hispida* (Diptera: Nycteribiidae) in Malaysia. Journal of Animal Ecology 40, 141–154.

[ref53] Mbu'u CM, Mbacham WF, Gontao P, Sado Kamdem SL, Nlôga AMN, Groschup MH, Wade A, Fischer K and Balkema-Buschmann A (2019) Henipaviruses at the interface between bats, livestock and human population in Africa. Vector-Borne and Zoonotic Diseases 19, 455–465. 30985268 10.1089/vbz.2018.2365

[ref54] McKee CD, Webb CT, Kosoy MY, Bai Y, Osikowicz LM, Suu-Ire R, Ntiamoa-Baidu Y, Cunningham AA, Wood JLN and Hayman DTS (2021) Manipulating vector transmission reveals local processes in bacterial communities of bats. Preprint. doi: 10.1101/2021.03.03.433743 PMC13215733 41622806

[ref55] Mickleburgh S, Waylen K and Racey P (2009) Bats as bushmeat: a global review. Oryx 43, 217–234.

[ref56] Mihaljevic JR (2012) Linking metacommunity theory and symbiont evolutionary ecology. Trends in Ecology & Evolution 27, 323–329. 22341499 10.1016/j.tree.2012.01.011

[ref57] Minh BQ, Schmidt HA, Chernomor O, Schrempf D, Woodhams MD, von Haeseler A and Lanfear R (2020) IQ-TREE 2: new models and efficient methods for phylogenetic inference in the genomic era. Molecular Biology and Evolution 37, 1530–1534. 32011700 10.1093/molbev/msaa015 PMC7182206

[ref58] Morse SF, Dick CW, Patterson BD and Dittmar K (2012) Some like it hot: evolution and ecology of novel endosymbionts in bat flies of cave-roosting bats (Hippoboscoidea, Nycterophiliinae). Applied and Environmental Microbiology 78, 8639–8649. 23042170 10.1128/AEM.02455-12 PMC3502899

[ref59] Morse SF, Bush SE, Patterson BD, Dick CW, Gruwell ME and Dittmar K (2013) Evolution, multiple acquisition, and localization of endosymbionts in bat flies (Diptera: Hippoboscoidea: Streblidae and Nycteribiidae). Applied and Environmental Microbiology 79, 2952–2961. 23435889 10.1128/AEM.03814-12 PMC3623134

[ref60] Mutere FA (1968) The breeding biology of the fruit bat ‘*Rousettus aegyptiacus*’ E. Geoffroy living at 0° 22’ S. Acta Tropica 25, 97–108. 4386699

[ref61] Nartey NAN (2015) Common parasites of fruit-eating bats in southern Ghana (MPhil thesis), University of Ghana, Accra, Ghana.

[ref62] Nguyen L-T, Schmidt HA, von Haeseler A and Minh BQ (2015) IQ-TREE: a fast and effective stochastic algorithm for estimating maximum-likelihood phylogenies. Molecular Biology and Evolution 32, 268–274. 25371430 10.1093/molbev/msu300 PMC4271533

[ref63] Nieberding CM and Olivieri I (2007) Parasites: proxies for host genealogy and ecology? Trends in Ecology & Evolution 22, 156–165. 17157954 10.1016/j.tree.2006.11.012

[ref64] Nieberding C, Morand S, Libois R and Michaux JR (2004) A parasite reveals cryptic phylogeographic history of its host. Proceedings of the Royal Society of London. Series B: Biological Sciences 271, 2559–2568. 10.1098/rspb.2004.2930 PMC1691906 15615681

[ref65] Nkoana TT, Kearney T and Markotter W (2023) Assessing age related cranial characteristics and morphometrics of the Egyptian rousette (*Rousettus aegyptiacus*) from Central Africa. Acta Chiropterologica 25, 169–181.

[ref66] Nováková E, Hypša V and Moran NA (2009) *Arsenophonus*, an emerging clade of intracellular symbionts with a broad host distribution. BMC Microbiology 9, 143. 19619300 10.1186/1471-2180-9-143 PMC2724383

[ref67] Ohemeng F, Lawson ET, Ayivor J, Leach M, Waldman L and Ntiamoa-Baidu Y (2017) Socio-cultural determinants of human–bat interactions in rural Ghana. Anthrozoös 30, 181–194.

[ref68] Oksanen J, Kindt R, Legendre P, O'Hara B, Simpson GL, Solymos P, Stevens MHH and Wagner H (2007) vegan: community ecology package. Retrieved from https://vegandevs.github.io/vegan/.

[ref69] Olival KJ, Dick CW, Simmons NB, Morales JC, Melnick DJ, Dittmar K, Perkins SL, Daszak P and DeSalle R (2013) Lack of population genetic structure and host specificity in the bat fly, *Cyclopodia horsfieldi*, across species of *Pteropus* bats in Southeast Asia. Parasites & Vectors 6, 231. 23924629 10.1186/1756-3305-6-231 PMC3750525

[ref70] Overal WL (1980) Host-relations of the batfly *Megistopoda aranea* (Diptera: Streblidae) in Panamá. The University of Kansas Science Bulletin 52, 1–20.

[ref71] Pawęska JT, Jansen van Vuren P, Storm N, Markotter W and Kemp A (2021) Vector competence of *Eucampsipoda africana* (Diptera: Nycteribiidae) for Marburg virus transmission in *Rousettus aegyptiacus* (Chiroptera: Pteropodidae). Viruses 13, 2226. 34835032 10.3390/v13112226 PMC8624361

[ref72] Peel AJ, Baker KS, Crameri G, Barr JA, Hayman DTS, Wright E, Broder CC, Fernández-Loras A, Fooks AR, Wang L-F, Cunningham AA and Wood JLN (2012) Henipavirus neutralising antibodies in an isolated island population of African fruit bats. PLoS ONE 7, e30346. 22253928 10.1371/journal.pone.0030346 PMC3257271

[ref73] Peel AJ, Sargan DR, Baker KS, Hayman DTS, Barr JA, Crameri G, Suu-Ire R, Broder CC, Lembo T, Wang L-F, Fooks AR, Rossiter SJ, Wood JLN and Cunningham AA (2013) Continent-wide panmixia of an African fruit bat facilitates transmission of potentially zoonotic viruses. Nature Communications 4, 2770. 10.1038/ncomms3770 PMC3836177 24253424

[ref74] Peel AJ, Baker KS, Hayman DTS, Suu-Ire R, Breed AC, Gembu G-C, Lembo T, Fernández-Loras A, Sargan DR, Fooks AR, Cunningham AA and Wood JLN (2016) Bat trait, genetic and pathogen data from large-scale investigations of African fruit bats, *Eidolon helvum*. Scientific Data 3, 160049. 27479120 10.1038/sdata.2016.49 PMC4968192

[ref75] Peel AJ, Wood JLN, Baker KS, Breed AC, de Carvalho A, Fernández-Loras A, Gabrieli HS, Gembu G-C, Kakengi VA, Kaliba PM, Kityo RM, Lembo T, Mba FE, Ramos D, Rodriguez-Prieto I, Suu-Ire R, Cunningham AA and Hayman DTS (2017) How does Africa's most hunted bat vary across the continent? Population traits of the straw-coloured fruit bat (*Eidolon helvum*) and its interactions with humans. Acta Chiropterologica 19, 77–92.

[ref76] Pernet O, Schneider BS, Beaty SM, LeBreton M, Yun TE, Park A, Zachariah TT, Bowden TA, Hitchens P, Ramirez CM, Daszak P, Mazet J, Freiberg AN, Wolfe ND and Lee B (2014) Evidence for henipavirus spillover into human populations in Africa. Nature Communications 5, 5342. 10.1038/ncomms6342 PMC4237230 25405640

[ref77] Qiu Y, Kajihara M, Nakao R, Mulenga E, Harima H, Hang'ombe BM, Eto Y, Changula K, Mwizabi D, Sawa H, Higashi H, Mweene A, Takada A, Simuunza M and Sugimoto C (2020) Isolation of *Candidatus* Bartonella rousetti and other bat-associated bartonellae from bats and their flies in Zambia. Pathogens 9, 469. 32545824 10.3390/pathogens9060469 PMC7350321

[ref78] Rajemison FI, Noroalintseheno LOS and Goodman SM (2017) Bat flies (Diptera: Nycteribiidae, Streblidae) parasitising *Rousettus madagascariensis* (Chiroptera: Pteropodidae) in the Parc National d'Ankarana, Madagascar: species diversity, rates of parasitism and sex ratios. African Entomology 25, 72–85.

[ref79] Ramanantsalama RV, Andrianarimisa A, Raselimanana AP and Goodman SM (2018) Rates of hematophagous ectoparasite consumption during grooming by an endemic Madagascar fruit bat. Parasites & Vectors 11, 330. 29859123 10.1186/s13071-018-2918-1 PMC5984742

[ref80] R Core Team (2023) R: a language and environment for statistical computing. Retrieved from https://www.r-project.org/.

[ref81] Reeves WK, Laverty TM, Gratton EM, Mushabati LM and Eiseb SJ (2020) New national records for *Cyclopodia greeffi greeffi* (Diptera: Nycteribiidae) from the Kunene Region, Namibia, Africa. Entomological News 129, 327–329.

[ref82] Richter HV and Cumming GS (2008) First application of satellite telemetry to track African straw-coloured fruit bat migration. Journal of Zoology 275, 172–176.

[ref83] Schwarz G (1978) Estimating the dimension of a model. The Annals of Statistics 6, 461–464.

[ref84] Seabloom EW, Borer ET, Gross K, Kendig AE, Lacroix C, Mitchell CE, Mordecai EA and Power AG (2015) The community ecology of pathogens: coinfection, coexistence and community composition. Ecology Letters 18, 401–415. 25728488 10.1111/ele.12418

[ref85] Speer KA, Luetke E, Bush E, Sheth B, Gerace A, Quicksall Z, Miyamoto M, Dick CW, Dittmar K, Albury N and Reed DL (2019) A fly on the cave wall: parasite genetics reveal fine-scale dispersal patterns of bats. Journal of Parasitology 105, 555–566. 31348717

[ref86] Špitalská E, Ševčík M, Peresh Y-Y and Benda P (2024) *Bartonella* in bat flies from the Egyptian fruit bat in the Middle East. Parasitology Research 123, 144. 38411931 10.1007/s00436-024-08165-6 PMC10899309

[ref87] Stribna T, Romportl D, Demjanovič J, Vogeler A, Tschapka M, Benda P, Horáček I, Juste J, Goodman SM and Hulva P (2019) Pan African phylogeography and palaeodistribution of rousettine fruit bats: ecogeographic correlation with Pleistocene climate vegetation cycles. Journal of Biogeography 46, 2336–2349.

[ref88] Szentiványi T, Heintz A-C, Markotter W, Wassef J, Christe P and Glaizot O (2023) Vector-borne protozoan and bacterial pathogen occurrence and diversity in ectoparasites of the Egyptian rousette bat. Medical and Veterinary Entomology 37, 189–194. 36625469 10.1111/mve.12639

[ref89] Sikes RS, Gannon WL and the Animal Care and Use Committee of the American Society of Mammalogists (2011) Guidelines of the American Society of Mammalogists for the use of wild mammals in research. Journal of Mammalogy 92, 235–253. 10.1093/jmammal/gyw078 PMC5909806 29692469

[ref90] Theodor O (1955) On the genus *Eucampsipoda* Kol. and *Dipseliopoda* n.g. (Nycteribiidae, Diptera). Parasitology 45, 195–229. 14370845 10.1017/s0031182000027578

[ref91] Theodor O (1957) The Nycteribiidae of the Ethiopian region and Madagascar. Parasitology 47, 457–543. 13504867 10.1017/s0031182000022162

[ref92] Theodor O (1967) An Illustrated Catalogue of the Rothschild Collection of Nycteribiidae (Diptera) in the British Museum (Natural History); with Keys and Short Descriptions for the Identification of Subfamilies, Genera, Species and Subspecies. London: British Museum (Natural History).

[ref93] Timen A, Koopmans MPG, Vossen ACTM, van Doornum GJJ, Günther S, van den Berkmortel F, Verduin KM, Dittrich S, Emmerich P, Osterhaus ADME, van Dissel JT and Coutinho RA (2009) Response to imported case of Marburg hemorrhagic fever, the Netherlands. Emerging Infectious Diseases 15, 1171–1175. 19751577 10.3201/eid1508.090051 PMC2815969

[ref94] Tukey JW (1949) Comparing individual means in the analysis of variance. Biometrics 5, 99–114. 18151955

[ref95] van Schaik J, Dekeukeleire D and Kerth G (2015) Host and parasite life history interplay to yield divergent population genetic structures in two ectoparasites living on the same bat species. Molecular Ecology 24, 2324–2335. 25809613 10.1111/mec.13171

[ref96] van Schaik J, Dekeukeleire D, Gazaryan S, Natradze I and Kerth G (2018a) Comparative phylogeography of a vulnerable bat and its ectoparasite reveals dispersal of a non-mobile parasite among distinct evolutionarily significant units of the host. Conservation Genetics 19, 481–494.

[ref97] Vellend M (2010) Conceptual synthesis in community ecology. The Quarterly Review of Biology 85, 183–206. 20565040 10.1086/652373

[ref98] Verrett TB, Webala PW, Patterson BD and Dick CW (2022) Remarkably low host specificity in the bat fly *Penicillidia fulvida* (Diptera: Nycteribiidae) as assessed by mitochondrial *COI* and nuclear 28*S* sequence data. Parasites & Vectors 15, 392. 36303252 10.1186/s13071-022-05516-z PMC9607801

[ref99] Vora NM, Osinubi MOV, Davis L, Abdurrahman M, Adedire EB, Akpan H, Aman-Oloniyo AF, Audu SW, Blau D, Dankoli RS, Ehimiyein AM, Ellison JA, Gbadegesin YH, Greenberg L, Haberling D, Hutson C, Idris JM, Kia GSN, Lawal M, Matthias SY, Mshelbwala PP, Niezgoda M, Ogunkoya AB, Ogunniyi AO, Okara GC, Olugasa BO, Ossai OP, Oyemakinde A, Person MK, Rupprecht CE, Saliman OA, Sani M, Sanni-Adeniyi OA, Satheshkumar PS, Smith TG, Soleye MO, Wallace RM, Yennan SK and Recuenco S (2020) Bat and lyssavirus exposure among humans in area that celebrates bat festival, Nigeria, 2010 and 2013. Emerging Infectious Diseases 26, 1399–1408. 32568051 10.3201/eid2607.191016 PMC7323560

[ref100] Wilkinson DA, Duron O, Cordonin C, Gomard Y, Ramasindrazana B, Mavingui P, Goodman SM and Tortosa P (2016) The bacteriome of bat flies (Nycteribiidae) from the Malagasy region: a community shaped by host ecology, bacterial transmission mode, and host-vector specificity. Applied and Environmental Microbiology 82, 1778–1788. 26746715 10.1128/AEM.03505-15 PMC4784053

[ref101] Wilson EB (1927) Probable inference, the law of succession, and statistical inference. Journal of the American Statistical Association 22, 209–212.

[ref102] Wirth T, Meyer A and Achtman M (2005) Deciphering host migrations and origins by means of their microbes. Molecular Ecology 14, 3289–3306. 16156803 10.1111/j.1365-294X.2005.02687.x

[ref103] Witsenburg F, Clément L, López-Baucells A, Palmeirim J, Pavlinić I, Scaravelli D, Ševčík M, Dutoit L, Salamin N, Goudet J and Christe P (2015) How a haemosporidian parasite of bats gets around: the genetic structure of a parasite, vector and host compared. Molecular Ecology 24, 926–940. 25641066 10.1111/mec.13071

[ref104] Zhu Q, Kosoy M, Olival KJ and Dittmar K (2014) Horizontal transfers and gene losses in the phospholipid pathway of *Bartonella* reveal clues about early ecological niches. Genome Biology and Evolution 6, 2156–2169. 25106622 10.1093/gbe/evu169 PMC4159011

