# Supplementary figures and images for: Ectoparasite and bacterial population genetics and community structure indicate extent of bat movement across an island chain
Source: Parasitology. 2024 May 24;151(7):708–21. doi: 10.1017/S0031182024000660 (PMC11474020; doi:10.1017/S0031182024000660)

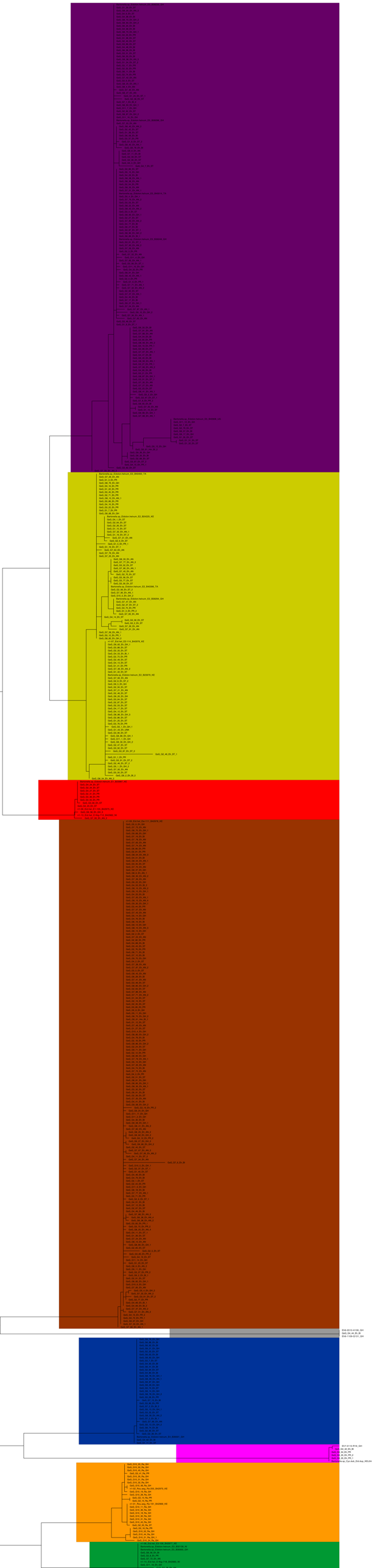

Supplement: McKee et al. supplementary material 1 — McKee et al. supplementary material [file S0031182024000660sup001.pdf]

ftsZ

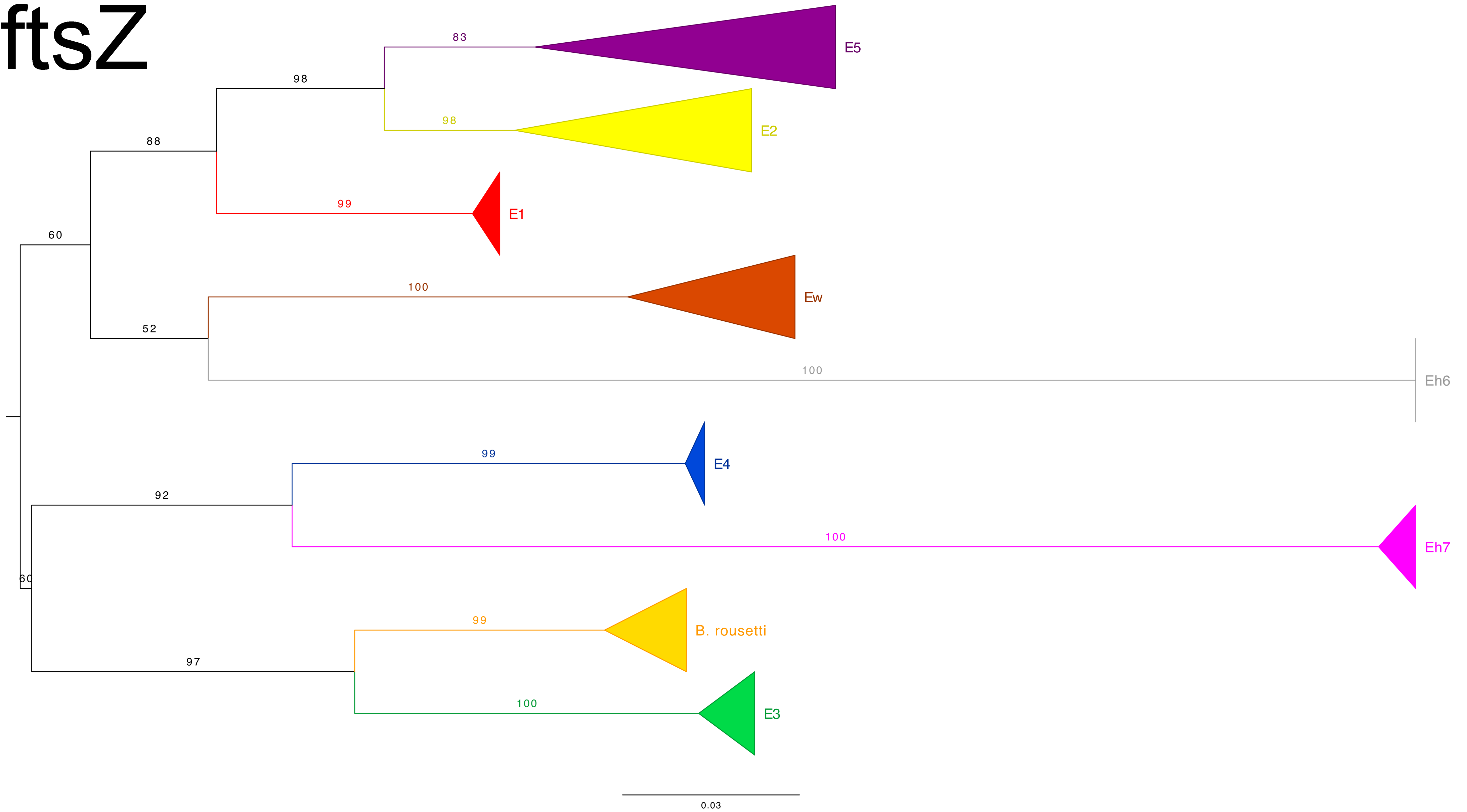

Supplement: McKee et al. supplementary material 2 — McKee et al. supplementary material [file S0031182024000660sup002.pdf]

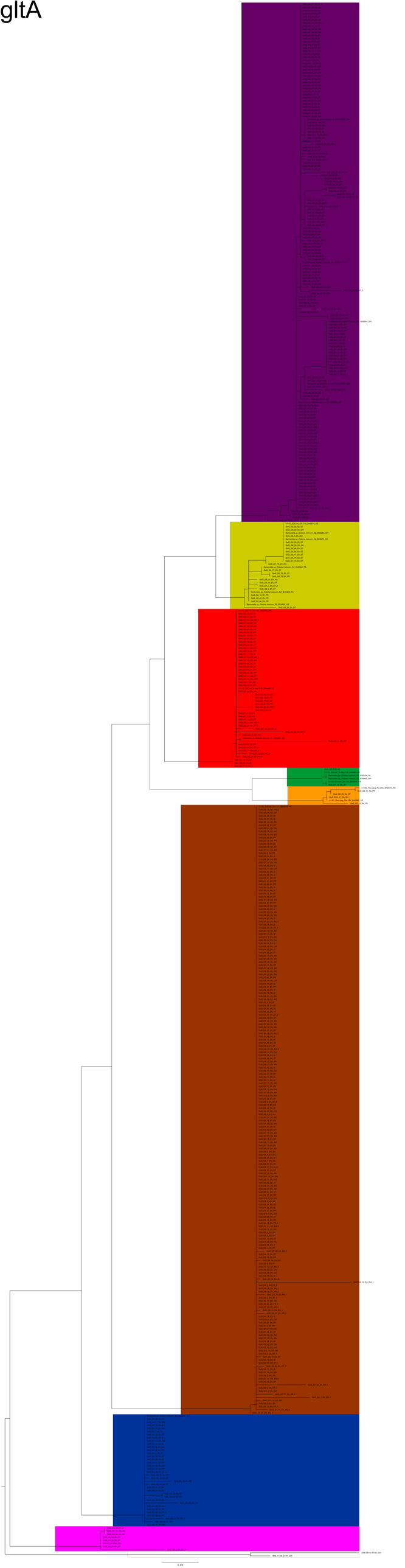

Supplement: McKee et al. supplementary material 3 — McKee et al. supplementary material [file S0031182024000660sup003.pdf]

# gltA

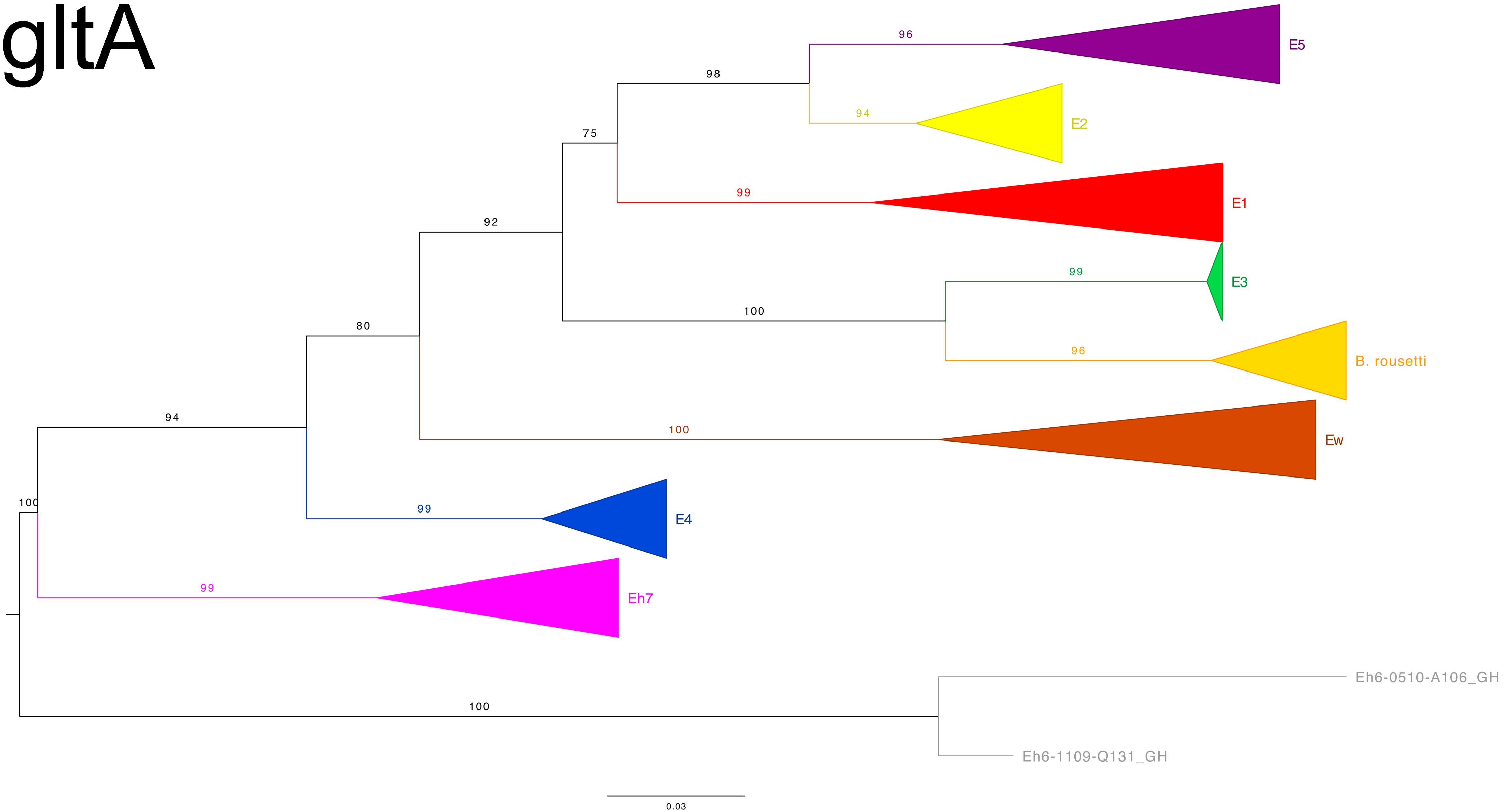

Supplement: McKee et al. supplementary material 4 — McKee et al. supplementary material [file S0031182024000660sup004.pdf]

# ITS

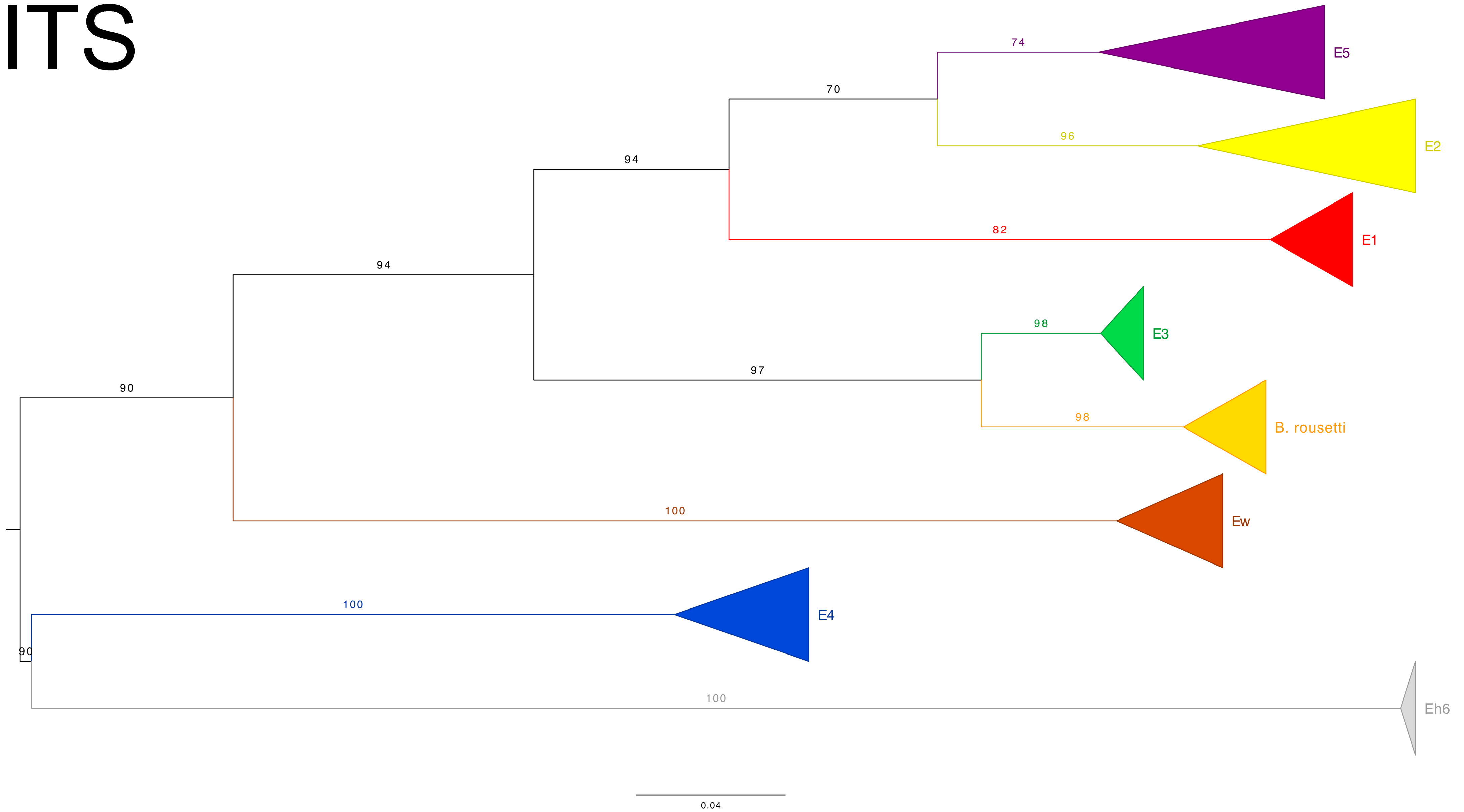

Supplement: McKee et al. supplementary material 6 — McKee et al. supplementary material [file S0031182024000660sup006.pdf]

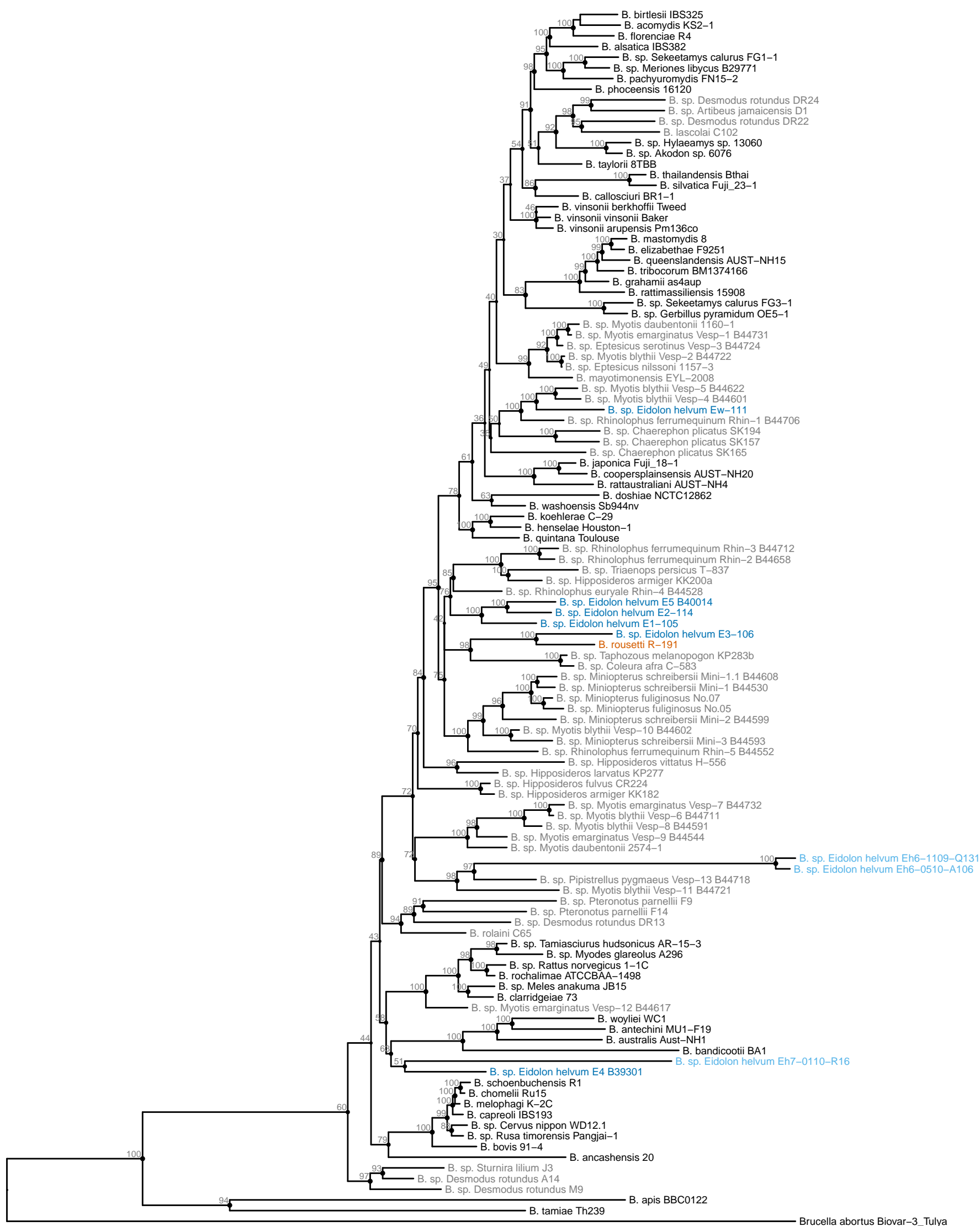

0.1

Supplement: McKee et al. supplementary material 7 — McKee et al. supplementary material [file S0031182024000660sup007.pdf]

### NMDS stress plot

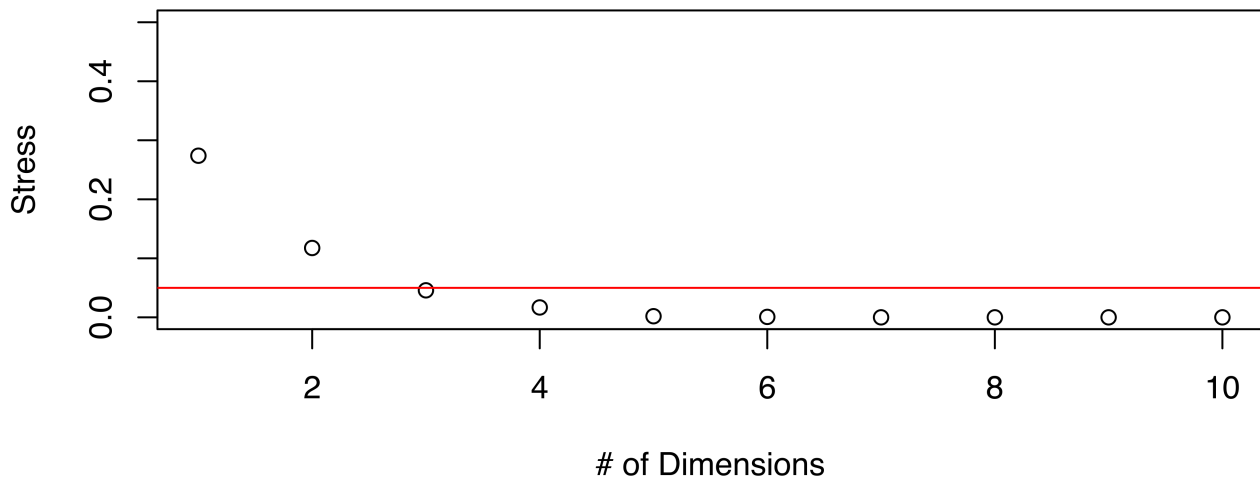

### Shepards diagram

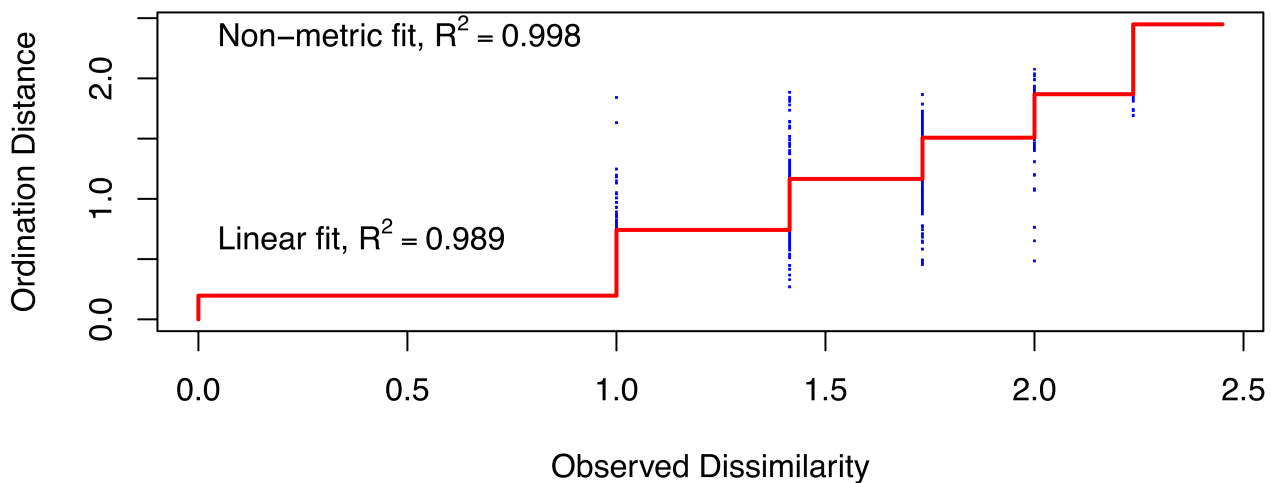

### NMDS ordination

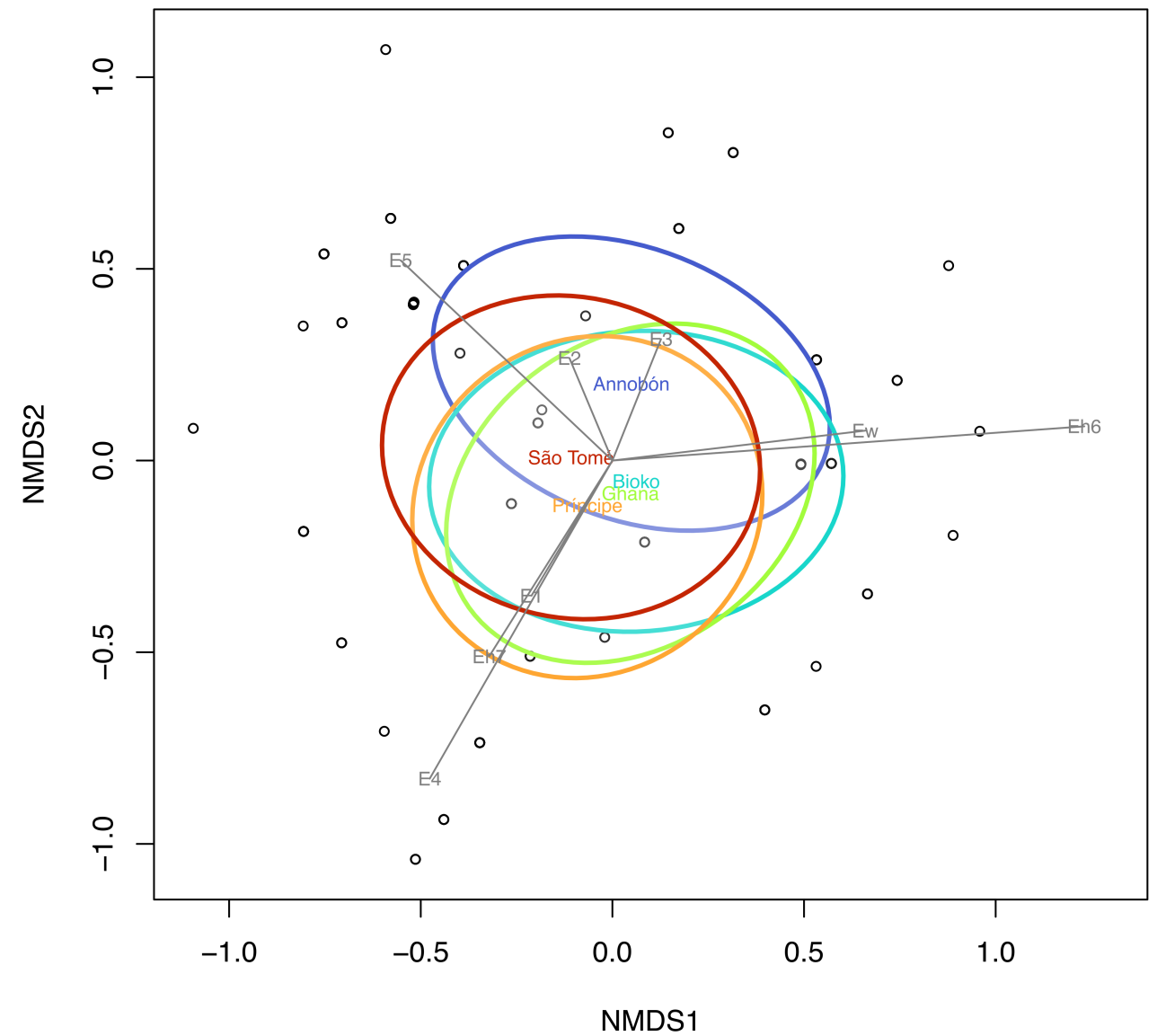

Supplement: McKee et al. supplementary material 8 — McKee et al. supplementary material [file S0031182024000660sup008.pdf]

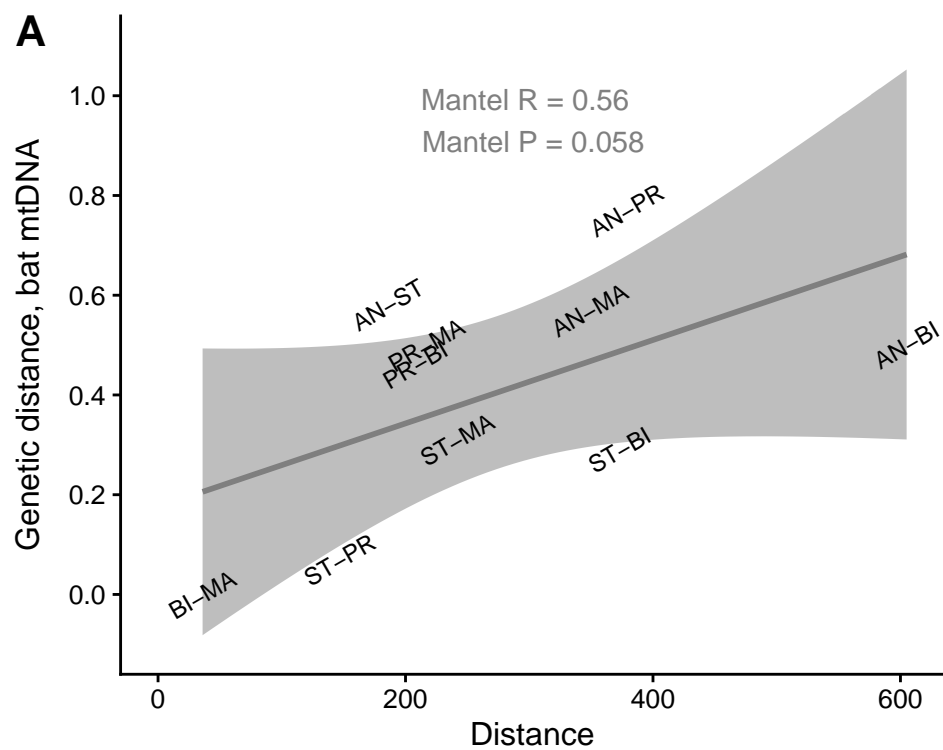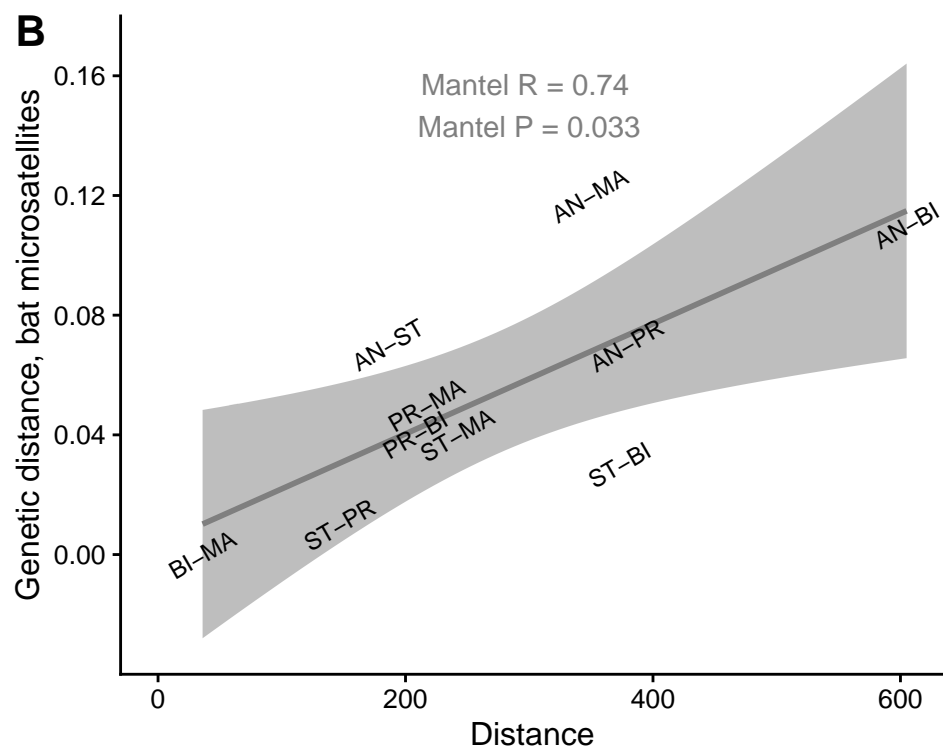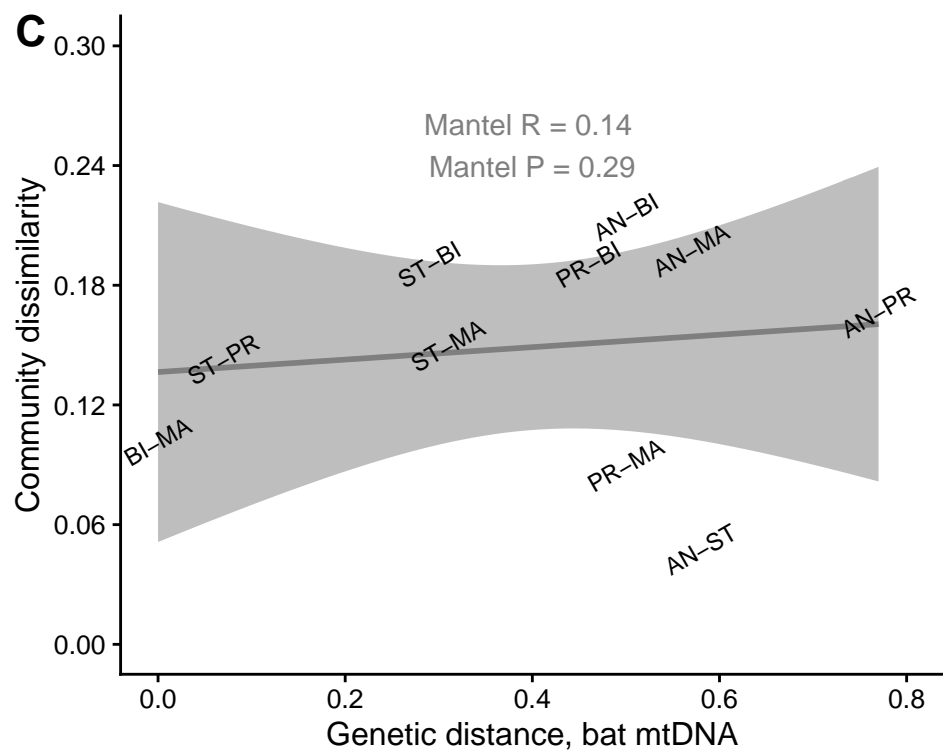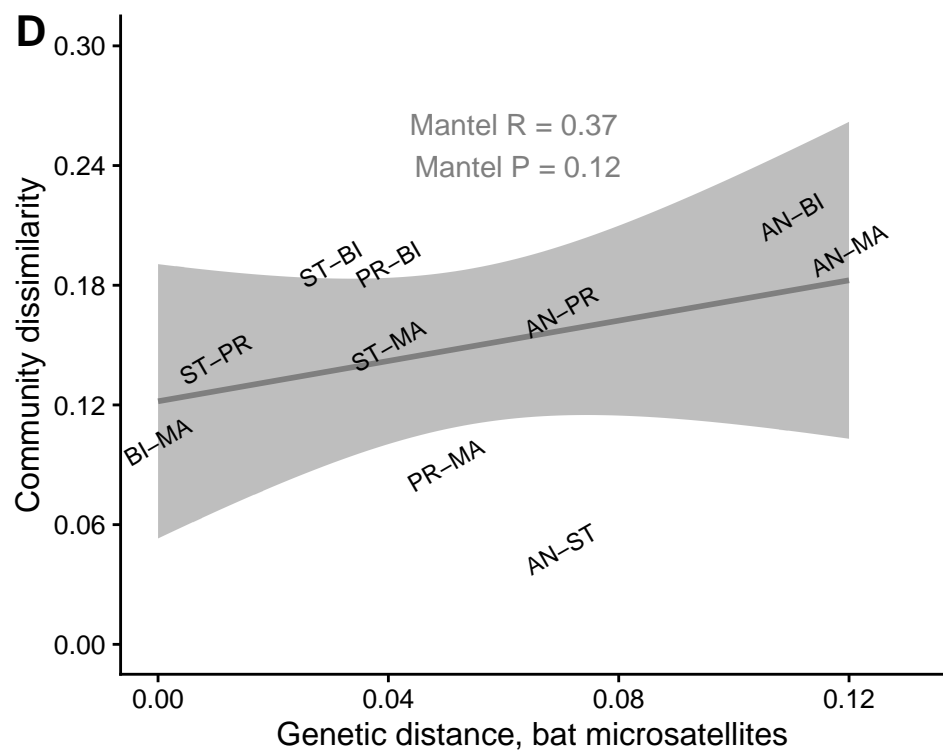

Supplement: McKee et al. supplementary material 9 — McKee et al. supplementary material [file S0031182024000660sup009.pdf]
