# Supplementary material for: Ectoparasite and bacterial population genetics and community structure indicate extent of bat movement across an island chain
Source: Parasitology. 2024 May 24;151(7):708–21. doi: 10.1017/S0031182024000660 (PMC11474020; doi:10.1017/S0031182024000660)
Supplement: McKee et al. supplementary material 10 — McKee et al. supplementary material [file S0031182024000660sup010.docx]

**Ectoparasite and bacterial population genetics and community structure indicate extent of bat movement across an island chain**

Clifton D. McKee, Alison J. Peel, David T. S. Hayman, Richard Suu-Ire, Yaa Ntiamoa-Baidu, Andrew A. Cunningham, James L. N. Wood, Colleen T. Webb, Michael Y. Kosoy

*Supplementary Material*

**Table S1.** Oligonucleotide primers used for haplotyping of bat flies and bacterial detection with conventional PCR amplification. Sequences designated [F] are forward primers and those designated [R] are reverse primers.

| **Target** | **Locus** | **PCR round** | **Primer sequence** | **Primer name** | **Product size (bp)** | **Reference** |
| --- | --- | --- | --- | --- | --- | --- |
| *Bartonella* | *ftsZ* | 1 | ATTAATCTGCAYCGGCCAGA [F] | Bfp1 | 885 | ^1^ |
|  |  |  | ACVGADACACGAATAACACC [R] | Bfp2 |  |  |
|  |  | 2 | ATATCGCGGAATTGAAGCC [F] | ftsZ R83 | 670 | ^2^ |
|  |  |  | CGCATAGAAGTATCATCCA [R] | ftsZ L83 |  |  |
| *Bartonella* | *gltA* | 1 | GCTATGTCTGCATTCTATCA [F] | CS443f | 767 | ^3, 4^ |
|  |  |  | GATCYTCAATCATTTCTTTCCA [R] | CS1210r |  |  |
|  |  | 2 | GGGGACCAGCTCATGGTGG [F] | BhCS781.p | 356 | ^3, 5^ |
|  |  |  | AATGCAAAAAGAACAGTAAACA [R] | BhCS1137.n |  |  |
| *Bartonella* | ITS | 1 | CTTCAGATGATGATCCCAAGCCTTCTGGCG [F] | 325s | 364-398 | ^6^ |
|  |  |  | GAACCGACGACCCCCTGCTTGCAAAGA [R] | 1100as |  |  |
| Arthropod mitochondrial DNA | 16S rRNA | 1 | TACGCTGTTATCCCTAA [F] | LR-J-13007 | 411 | ^7-9^ |
|  |  |  | CGCCTGTTTATCAAAAACAT [R] | LR-N-13398 |  |  |
| Arthropod mitochondrial DNA | *cytb* | 1 | AGGRCAAATATCATTTTGAG [F] | A5 | 387 | ^10^ |
|  |  |  | AAATATCATTCTGGTTGAATATG [R] | B1.1 |  |  |
| *Enterobacteriales* | 16S rRNA | 1 | GGGTTGTAAAGTACTTTCAGTCGT [F] | ArsF | 575 | ^11^ |
|  |  |  | CCTYTATCTCTAAAGGMTTCGCTGGATG [R] | ArsR3 |  |  |

References: ^1^Zeaiter *et al.* (2002)⁠; ^2^Colborn *et al.* (2010); ^3^Birtles & Raoult (1996)⁠; ^4^Gundi *et al.* (2012)⁠; ^5^Norman *et al.* (1995); ^6^Diniz *et al.* (2007)⁠; ^7^Simon *et al.* (1994); ^8^Kambhampati & Smith (1995); ^9^Szalanski *et al.* (2004); ^10^Dittmar de la Cruz & Whiting (2003); ^11^Duron *et al.* (2008).

**Table S2.** Thermocycler protocols used for conventional PCR amplification.

| **Target** | **Locus** | **PCR round** | **Thermal program** |
| --- | --- | --- | --- |
| *Bartonella* | *ftsZ* | 1 | 95°C 4:00, (95°C 0:30, 55°C 0:30, 72°C 1:00)x40, 72°C 10:00, 4°C ∞ |
|  |  | 2 | 95°C 4:00, (95°C 0:30, 55°C 0:30, 72°C 1:00)x40, 72°C 10:00, 4°C ∞ |
| *Bartonella* | *gltA* | 1 | 95°C 2:00, (95°C 0:30, 48°C 0:30, 72°C 2:00)x40, 72°C 7:00, 4°C ∞ |
|  |  | 2 | 95°C 3:00, (95°C 0:30, 55°C 0:30, 72°C 0:30)x40, 72°C 7:00, 4°C ∞ |
| *Bartonella* | ITS | 1 | 95°C 3:00, (95°C 0:30, 66°C 0:30, 72°C 0:30)x55, 72°C 5:00, 4°C ∞ |
| Arthropod mitochondrial DNA | 16S rRNA | 1 | 95°C 3:00, (95°C 0:45, 46°C 0:45, 72°C 0:45)x55, 72°C 7:00, 4°C ∞ |
| Arthropod mitochondrial DNA | *cytb* | 1 | 95°C 12:00, (95°C 0:30, 40°C 0:30, 72°C 2:00)x55, 72°C 7:00, 4°C ∞ |
| *Enterobacteriales* | 16S rRNA | 1 | 95°C 2:00, (95°C 0:30, 52°C 0:30, 72°C 1:30)x55, 72°C 5:00, 4°C ∞ |

**Table S3.** Counts of bat fly and bat fly *Enterobacterales* symbiont haplotypes detected at each sampling location. Counts were used for calculation of relative abundance in Figure 3B,D,F.

| **Locus** | **Bat fly species** | **Haplotype** | **Sampling location** | **Counts of bat flies with mitochondrial or bacterial symbiont haplotype** |
| --- | --- | --- | --- | --- |
| ectoparasite mitochondrial 16S rRNA | *C. greefi* | 1 | Annobón | 81 |
|  |  |  | Bioko | 38 |
|  |  |  | Ghana | 41 |
|  |  |  | Príncipe | 55 |
|  |  |  | São Tomé | 93 |
|  | *E. africana* | 1 | Ghana | 25 |
|  |  |  | Nigeria | 7 |
|  |  | 2 | Príncipe | 10 |
|  |  |  | São Tomé | 1 |
|  | *D. biannulata* | 1 | Ghana | 1 |
| ectoparasite mitochondrial *cytb* | *C. greefi* | 1 | Annobón | 3 |
|  |  |  | Bioko | 10 |
|  |  |  | Ghana | 25 |
|  |  |  | Príncipe | 9 |
|  |  |  | São Tomé | 32 |
|  |  | 2 | Annobón | 8 |
|  | *E. africana* | 1 | Ghana | 15 |
|  |  | 2 | Ghana | 4 |
|  |  | 3 | Ghana | 3 |
|  |  | 4 | Ghana | 18 |
|  |  | 5 | Príncipe | 9 |
|  |  |  | São Tomé | 1 |
| *Enterobacterales* symbiont 16S rRNA | *C. greefi* | 1 | Annobón | 1 |
|  |  |  | Ghana | 12 |
|  |  |  | Príncipe | 3 |
|  |  |  | São Tomé | 5 |
|  | *E. africana* | 1 | Ghana | 11 |
|  |  | 2 | Príncipe | 3 |
|  |  |  | São Tomé | 1 |

**Table S4.** *Bartonella* infection prevalence in bat flies across sampling years by species. Samples were considered positive for *Bartonella* bacteria if one or more genetic markers produced a sequence confirmed as *Bartonella*. Binomial 95% confidence intervals for prevalence were estimated using Wilson score intervals. Differences for *Dipseliopoda biannulata* were not exampled because only one specimen was collected in 2016.

| **Bat host species** | **Bat fly species** | **Sampling year** | **Samples** | ***Bartonella* positive** | **Prevalence** |
| --- | --- | --- | --- | --- | --- |
| *Eidolon helvum* | *Cyclopodia greefi* | 2009 | 49 | 40 | 0.82 (0.69–0.9) |
|  |  | 2010 | 551 | 436 | 0.79 (0.76–0.82) |
|  |  | 2012 | 18 | 14 | 0.78 (0.55–0.91) |
|  |  | 2016 | 90 | 76 | 0.84 (0.76–0.91) |
| *Rousettus aegyptiacus* | *Eucampsipoda africana* | 2010 | 11 | 5 | 0.45 (0.21–0.72) |
|  |  | 2012 | 22 | 7 | 0.32 (0.16–0.53) |
|  |  | 2016 | 23 | 12 | 0.52 (0.33–0.71) |

**Table S5.** Counts of *Bartonella* genogroups detected in bat flies across locations. Counts are based on the presence of sequences representing a given genogroup from any of three genetic loci used for detection (ITS, *ftsZ*, *gltA*). Counts were used for calculation of relative abundance in Figure 4A and community dissimilarity in Figure 5.

| **Bat host species** | **Bat fly species** | **Location** | **E1** | **E2** | **E3** | **E4** | **E5** | **Ew** | **Eh6** | **Eh7** | ***B. rousetti*** |
| --- | --- | --- | --- | --- | --- | --- | --- | --- | --- | --- | --- |
| *E. helvum* | *C. greefi* | Ghana | 3 | 16 | 11 | 36 | 30 | 60 | 0 | 0 | 0 |
|  |  | Bioko | 5 | 3 | 7 | 30 | 36 | 60 | 1 | 2 | 0 |
|  |  | Príncipe | 13 | 16 | 1 | 21 | 19 | 27 | 0 | 2 | 0 |
|  |  | São Tomé | 17 | 43 | 6 | 29 | 48 | 45 | 0 | 4 | 0 |
|  |  | Annobón | 13 | 25 | 7 | 10 | 59 | 69 | 0 | 1 | 0 |
| *R. aegyptiacus* | *E. africana* | Ghana | 0 | 0 | 0 | 0 | 0 | 0 | 0 | 0 | 19 |
|  |  | Príncipe | 0 | 0 | 0 | 0 | 0 | 0 | 0 | 0 | 4 |
|  |  | São Tomé | 0 | 0 | 0 | 0 | 0 | 0 | 0 | 0 | 1 |

**Table S6.** Age distribution of *E. helvum* populations sampled for bat flies. Ages are abbreviated N – neonate, J – juvenile, SI – sexually immature adult, and A – sexually mature adult. Counts were used for calculation of relative abundance in Figure 4C. Note that many individuals captured on Bioko island in May 2010 were free-flying dependent young that were less than two months old (Peel *et al.*, 2017; below the age cutoff for juveniles), so are thus lumped with other neonates.

| **Location** | **Years sampled** | **N** | **J** | **SI** | **A** | **Total** |
| --- | --- | --- | --- | --- | --- | --- |
| Annobón | 2010 | 1 | 0 | 69 | 132 | 202 |
| Bioko | 2010 | 84 | 0 | 4 | 17 | 105 |
| Príncipe | 2010 | 0 | 10 | 11 | 40 | 61 |
| São Tomé | 2010 | 26 | 0 | 15 | 61 | 102 |
| Ghana | 2009, 2012, 2016 | 20 | 63 | 406 | 1220 | 1709 |

**Table S7.** Distance measures for sampled populations. Physical distance is measured in kilometers between islands and the mainland, considering Ghana as a representative population for the mainland as in Figure 2B. *Bartonella* community dissimilarity is calculated as one minus the Spearman rank correlation between counts of *Bartonella* genogroups across locations. Genetic distances for *E. helvum* across locations are recorded as Slatkin’s linearized ϕ_ST_ (ϕ_ST_/(1 - ϕ_ST_)) for mitochondrial *cytb* sequences and F_ST_ (F_ST_/(1 - F_ST_)) for microsatellites taken from Peel *et al.* (2013).

| **Comparison** | **Physical distance** | ***Bartonella* community dissimilarity** | ***E. helvum* genetic distance, mtDNA** | ***E. helvum* genetic distance, microsatellites** |
| --- | --- | --- | --- | --- |
| Bioko–Mainland | 35.9 | 0.1 | 0 | 0 |
| Príncipe–Mainland | 217.2 | 0.09 | 0.5 | 0.05 |
| São Tomé–Mainland | 242.4 | 0.15 | 0.31 | 0.04 |
| Annobón–Mainland | 349.3 | 0.2 | 0.57 | 0.12 |
| Príncipe–Bioko | 207.6 | 0.19 | 0.46 | 0.04 |
| São Tomé–Bioko | 372.7 | 0.19 | 0.29 | 0.03 |
| Annobón–Bioko | 604.6 | 0.21 | 0.5 | 0.11 |
| São Tomé–Príncipe | 147.1 | 0.14 | 0.07 | 0.01 |
| Annobón–Príncipe | 378.8 | 0.17 | 0.77 | 0.07 |
| Annobón–São Tomé | 185.5 | 0.05 | 0.58 | 0.07 |

**Table S8.** Bat fly specimens tested for bacterial symbionts by PCR. The number of positive specimens based on confirmed *Enterobacterales* sequences is recorded. Binomial 95% confidence intervals for prevalence were estimated using Wilson score intervals.

| **Bat fly species** | **Location** | **Samples** | **Tested** | **Symbiont positive** | **Prevalence** |
| --- | --- | --- | --- | --- | --- |
| *C. greefi* | Ghana | 158 | 70 | 12 | 0.17 (0.1–0.28) |
|  | Bioko | 176 | 138 | 0 | 0 (0–0.03) |
|  | Príncipe | 81 | 74 | 3 | 0.04 (0.01–0.11) |
|  | São Tomé | 165 | 135 | 5 | 0.04 (0.02–0.08) |
|  | Annobón | 131 | 95 | 1 | 0.01 (0–0.06) |
| *E. africana* | Ghana | 44 | 43 | 11 | 0.26 (0.15–0.4) |
|  | Príncipe | 10 | 10 | 3 | 0.3 (0.11–0.6) |
|  | São Tomé | 1 | 1 | 1 | 1 (0.21–1) |

**Figure S1.** Maximum likelihood phylogenetic tree of *Bartonella* cell division protein gene (*ftsZ*) sequences produced from a 638 bp alignment of 515 sequences. The best model of sequence evolution according to IQ-Tree was HKY+F+G4 based on BIC. The tree was rooted at the midpoint and separate genogroups are highlighted in different colors: E1 – red, E2 – yellow, E3 – green, E4 – blue, E5 – purple, Ew – brown, Eh6 – gray, Eh7 – magenta, *B. rousetti* – orange. Abbreviations in sequence names: Eh – *Eidolon helvum*, Hm – *Hypsignathus monstrosus*, Ra – *Rousettus aegyptiacus*, AN – Annobón, BI – Bioko, GH – Ghana, PR – Príncipe, ST – São Tomé.

**Figure S2.** Collapsed version of the *Bartonella* *ftsZ* maximum likelihood tree showing separate genogroups and branch support. Colors for genotypes are the same as in Figure S1. Branch support values for each genogroup cluster are shown along the branch to the left of each node.

**Figure S3.** Maximum likelihood phylogenetic tree of *Bartonella* citrate synthase gene (*gltA*) sequences produced from a 301 bp alignment of 500 sequences. The best model of sequence evolution according to IQ-Tree was TIM3+F+G4 based on BIC. The tree was rooted at the midpoint and separate genogroups are highlighted in different colors: E1 – red, E2 – yellow, E3 – green, E4 – blue, E5 – purple, Ew – brown, Eh6 – gray, Eh7 – magenta, *B. rousetti* – orange. Abbreviations in sequence names: Eh – *Eidolon helvum*, Hm – *Hypsignathus monstrosus*, Ra – *Rousettus aegyptiacus*, AN – Annobón, BI – Bioko, GH – Ghana, PR – Príncipe, ST – São Tomé.

**Figure S4.** Collapsed version of the *Bartonella* *gltA* maximum likelihood tree showing separate genogroups and branch support. Colors for genotypes are the same as in Figure S1. Branch support values for each genogroup cluster are shown along the branch to the left of each node.

**Figure S5.** Maximum likelihood phylogenetic tree of *Bartonella* 16S–23S ribosomal RNA intergenic spacer region (ITS) sequences produced from a 522 bp alignment of 542 sequences. The best model of sequence evolution according to IQ-Tree was HKY+F+R3 based on BIC. The tree was rooted at the midpoint and separate genogroups are highlighted in different colors: E1 – red, E2 – yellow, E3 – green, E4 – blue, E5 – purple, Ew – brown, Eh6 – gray, Eh7 – magenta, *B. rousetti* – orange. Abbreviations in sequence names: Eh – *Eidolon helvum*, Hm – *Hypsignathus monstrosus*, Ra – *Rousettus aegyptiacus*, AN – Annobón, BI – Bioko, GH – Ghana, PR – Príncipe, ST – São Tomé.

**Figure S6.** Collapsed version of the *Bartonella* ITS maximum likelihood tree showing separate genogroups and branch support. Colors for genotypes are the same as in Figure S1. Branch support values for each genogroup cluster are shown along the branch to the left of each node.

**Figure S7.** Maximum likelihood phylogenetic tree of concatenated *Bartonella* *ftsZ* and *gltA* sequences produced from a 1247 bp alignment (891 bp *ftsZ*, 356 bp *gltA*) of 114 sequences. The best model of sequence evolution according to IQ-Tree was TVM+F+R7 based on BIC. The tree was rooted at the midpoint and bootstrap branch support values are shown in gray next to branches. Names of *Bartonella* species/strains previously obtained from bats are colored gray, genogroups previously obtained from *E. helvum* or *C. greefi* are colored dark blue, genogroups from *R. aegyptiacus* or *E. africana* are colored orange, and names of new genogroups from *E. helvum*/*C. greefi* are colored light blue.

**Figure S8.** Nonmetric multidimensional scaling (NMDS) ordination of *Bartonella* community composition in individual bat flies tested from *E. helvum*. Ordination was performed using a Euclidean distance matrix based on presence/absence of *Bartonella* genogroups in individual bat flies from each sampling location. The scree plot (top left) shows the reduction in stress with increasing number of dimensions for NMDS, with the red line at 0.05 showing the recommended cutoff. After 250 random starts to the NMDS using three dimensions, the stable solution had excellent fit between the observed dissimilarity and ordination distances (bottom left). The stable ordination solution (right) showed that in two dimensions, different *Bartonella* genogroups had stronger weighting (as indicated by length of lines from the origin). *Bartonella* communities were largely similar across sampling locations, shown by overlapping 95% confidence interval ordination ellipses and closely clustered centroids for each sampling location.

**Figure S9.** Correlations between genetic data from *E. helvum* populations, physical distance between sampling locations, and *Bartonella* community dissimilarity. Mantel tests based on Pearson’s correlation were performed with 119 permutations (the complete set for the 5x5 matrices). (A) Relationship between physical distance and mtDNA genetic distances, Slatkin’s linearized ϕ_ST_ (ϕ_ST_/(1 - ϕ_ST_)) for *cytb* sequences. (B) Relationship between physical distance and genetic distances for microsatellites, Slatkin’s linearized F_ST_ (F_ST_/(1 - F_ST_)). (C) Relationship between genetic distances from mtDNA and *Bartonella* community dissimilarity. (D) Relationship between genetic distances from microsatellites and *Bartonella* community dissimilarity. All values are recorded in Table S7. Locations are abbreviated AN – Annobón, BI – Bioko, MA – mainland (Ghana), PR – Príncipe, and ST - São Tomé.

**References**

**Birtles, R. J. and Raoult, D.** (1996). Comparison of partial citrate synthase gene (*gltA*) sequences for phylogenetic analysis of *Bartonella* species. *International Journal of Systematic and Evolutionary Microbiology* **46**, 891–897. doi: 10.1099/00207713-46-4-891.

**Colborn, J. M., Kosoy, M. Y., Motin, V. L., Telepnev, M. V., Valbuena, G., Myint, K. S., Fofanov, Y., Putonti, C., Feng, C. and Peruski, L.** (2010). Improved detection of *Bartonella* DNA in mammalian hosts and arthropod vectors by real-time PCR using the NADH dehydrogenase gamma subunit (*nuoG*). *Journal of Clinical Microbiology* **48**, 4630–4633. doi: 10.1128/jcm.00470-10.

**Diniz, P. P. V. D. P., Maggi, R. G., Schwartz, D. S., Cadenas, M. B., Bradley, J. M., Hegarty, B. and Breitschwerdt, E. B.** (2007). Canine bartonellosis: serological and molecular prevalence in Brazil and evidence of co-infection with *Bartonella henselae* and *Bartonella vinsonii* subsp. *berkhoffii*. *Veterinary Research* **38**, 697–710. doi: 10.1051/vetres:2007023.

**Dittmar de la Cruz, K. and Whiting, M. F.** (2003). Genetic and phylogeographic structure of populations of *Pulex simulans* (Siphonaptera) in Peru inferred from two genes (*CytB* and *CoII*). *Parasitology Research* **91**, 55–59. doi: 10.1007/s00436-003-0879-5.

**Duron, O., Bouchon, D., Boutin, S., Bellamy, L., Zhou, L., Engelstädter, J. and Hurst, G. D.** (2008). The diversity of reproductive parasites among arthropods: *Wolbachia* do not walk alone. *BMC Biology* **6**, 27. doi: 10.1186/1741-7007-6-27.

**Gundi, V. A. K. B., Billeter, S. A., Rood, M. P. and Kosoy, M. Y.** (2012). *Bartonella* spp. in rats and zoonoses, Los Angeles, California, USA. *Emerging Infectious Diseases* **18**, 631–633. doi: 10.3201/eid1804.110816.

**Kambhampati, S. and Smith, P. T.** (1995). PCR primers for the amplification of four insect mitochondrial gene fragments. *Insect Molecular Biology* **4**, 233–236. doi: 10.1111/j.1365-2583.1995.tb00028.x.

**Norman, A. F., Regnery, R., Jameson, P., Greene, C. and Krause, D. C.** (1995). Differentiation of *Bartonella*-like isolates at the species level by PCR-restriction fragment length polymorphism in the citrate synthase gene. *Journal of Clinical Microbiology* **33**, 1797–1803. doi: 10.1128/jcm.33.7.1797-1803.1995.

**Peel, A. J., Sargan, D. R., Baker, K. S., Hayman, D. T. S., Barr, J. A., Crameri, G., Suu-Ire, R., Broder, C. C., Lembo, T., Wang, L.-F., Fooks, A. R., Rossiter, S. J., Wood, J. L. N. and Cunningham, A. A.** (2013). Continent-wide panmixia of an African fruit bat facilitates transmission of potentially zoonotic viruses. *Nature Communications* **4**, 2770. doi: 10.1038/ncomms3770.

**Peel, A. J., Wood, J. L. N., Baker, K. S., Breed, A. C., de Carvalho, A., Fernández-Loras, A., Gabrieli, H. S., Gembu, G.-C., Kakengi, V. A., Kaliba, P. M., Kityo, R. M., Lembo, T., Mba, F. E., Ramos, D., Rodriguez-Prieto, I., Suu-Ire, R., Cunningham, A. A. and Hayman, D. T. S.** (2017). How does Africa’s most hunted bat vary across the continent? Population traits of the straw-coloured fruit bat (*Eidolon helvum*) and its interactions with humans. *Acta Chiropterologica* **19**, 77–92. doi: 10.3161/15081109ACC2017.19.1.006.

**Simon, C., Frati, F., Beckenbach, A., Crespi, B., Liu, H. and Flook, P.** (1994). Evolution, weighting, and phylogenetic utility of mitochondrial gene sequences and a compilation of conserved polymerase chain reaction primers. *Annals of the Entomological Society of America* **87**, 651–701. doi: 10.1093/aesa/87.6.651.

**Szalanski, A. L., Austin, J. W., Scheffrahn, R. H. and Messenger, M. T.** (2004). Molecular diagnostics of the Formosan subterranean termite (Isoptera: Rhinotermitidae). *Florida Entomologist* **87**, 145–151. doi: 10.1653/0015-4040(2004)087[0145:MDOTFS]2.0.CO;2.

**Zeaiter, Z., Liang, Z. and Raoult, D.** (2002). Genetic classification and differentiation of *Bartonella* species based on comparison of partial *ftsZ* gene sequences. *Journal of Clinical Microbiology* **40**, 3641–3647. doi: 10.1128/jcm.40.10.3641-3647.2002.
