# Supplementary material for: Ectoparasite and bacterial population genetics and community structure indicate extent of bat movement across an island chain
Source: Parasitology. 2024 May 24;151(7):708–21. doi: 10.1017/S0031182024000660 (PMC11474020; doi:10.1017/S0031182024000660)

# ITS

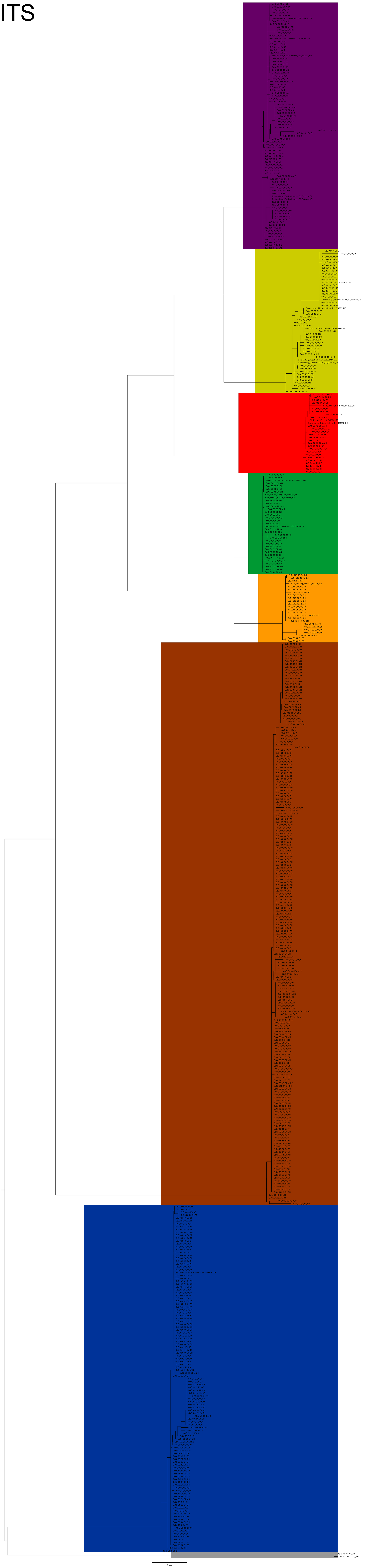

Supplement: McKee et al. supplementary material 5 — McKee et al. supplementary material [file S0031182024000660sup005.pdf]
